# MutS‐Homolog2 silencing generates tetraploid meiocytes in tomato (*Solanum lycopersicum*)

**DOI:** 10.1002/pld3.17

**Published:** 2018-01-02

**Authors:** Supriya Sarma, Arun Kumar Pandey, Kapil Sharma, Maruthachalam Ravi, Yellamaraju Sreelakshmi, Rameshwar Sharma

**Affiliations:** ^1^ Repository of Tomato Genomics Resources Department of Plant Sciences School of Life Sciences University of Hyderabad Hyderabad India; ^2^ School of Biology Indian Institute of Science Education and Research Thiruvananthapuram Kerala India; ^3^ Present address: Centre for Cellular and Molecular Biology Hyderabad India; ^4^ Present address: International Crops Research Institute for the Semi‐Arid Tropics (ICRISAT) Hyderabad India

**Keywords:** cytokinesis, meiosis, mitosis, MutS‐Homolog2, polyploidy, *Solanum lycopersicum*, tetraploid meiocytes, tomato

## Abstract

MSH2 is the core protein of MutS‐homolog family involved in recognition and repair of the errors in the DNA. While other members of MutS‐homolog family reportedly regulate mitochondrial stability, meiosis, and fertility, MSH2 is believed to participate mainly in mismatch repair. The search for polymorphism in *MSH2* sequence in tomato accessions revealed both synonymous and nonsynonymous SNPs; however, SIFT algorithm predicted that none of the SNPs influenced MSH2 protein function. The silencing of *MSH2* gene expression by RNAi led to phenotypic abnormalities in highly silenced lines, particularly in the stamens with highly reduced pollen formation. *MSH2* silencing exacerbated formation of UV‐B‐induced thymine dimers and blocked light‐induced repair of the dimers. The *MSH2* silencing also affected the progression of male meiosis to a varying degree with either halt of meiosis at zygotene stage or formation of diploid tetrads. The immunostaining of male meiocytes with centromere localized CENPC (centromere protein C) antibody showed the presence of 48 univalent along with 24 bivalent chromosomes suggesting abnormal tetraploid meiosis. The mitotic cells of root tips of silenced lines showed diploid nuclei but lacked intervening cell plates leading to cells with syncytial nuclei. Thus, we speculate that tetraploid pollen mother cells may have arisen due to the fusion of syncytial nuclei before the onset of meiosis. It is likely that in addition to mismatch repair (MMR), MSH2 may have an additional role in regulating ploidy stability.

## 1 INTRODUCTION

The survival of the living organisms is dependent on the precise and error‐free replication of DNA. In addition, normal metabolic activity and environmental factors such as radiation also cause damage to the DNA. The fidelity of the DNA is ensured by a cohort of mechanisms that detects and eliminates mistakes occurring during DNA replication. At least five major repair pathways are known to operate in higher plants: base excision repair, nucleotide excision repair, mismatch repair (MMR), and double‐strand break repair comprising of homologous recombination repair and nonhomologous end joining (Guarné et al., 2004; Jacobs & Schär, 2012; Reardon & Sancar, 2005; Takata et al., 1998). Among these, MMR is a major pathway that corrects base–base and insertion–deletion mismatches generated during DNA replication or mutagenesis (Dowen, Putnam, & Kolodner, 2010; Srivatsan, Bowen, & Kolodner, 2014).

MMR is a highly conserved pathway that exists in all living organisms. The MMR process consists of three key steps: mismatch recognition, excision, and resynthesis (Antony & Hingorani, 2003; Ban, Junop, & Yang, 1999; Guarné et al., 2004). The first step of the mismatch recognition in eukaryotes is carried out by the homologs of prokaryotic MutS proteins, namely MSH protein subunits. There are eight homologs of MutS in eukaryotes: MSH1 to MSH8, of which MSH7 is found only in plants (Culligan & Hays, 2000) and MSH8 in Euglenozoa (Sachadyn, 2010). The MSH proteins recognize mismatches as heterodimers, where MutSα (MSH2‐MSH6) repairs base–base mismatches or 1–2 nucleotide insertion–deletion loops (Acharya et al., 1996; Genschel, Littman, Drummond, & Modrich, 1998), while MutSβ (MSH2‐MSH3) recognizes larger, up to 14 nucleotide insertion–deletion loops (Marti, Kunz, & Fleck, 2002; Modrich, 1991). Plants form an additional heterodimeric complex known as MutSγ (MSH2–MSH7) (Culligan & Hays, 2000) which recognizes some base–base mismatches and reportedly plays a role in meiotic recombination (Lloyd, Milligan, Langridge, & Able, 2007). The binding of MutSα/β to mismatched DNA initiates conformational changes in MutS recruiting MutL complex followed by activation of exonuclease1/PCNA. The gap generated by DNA excision is filled by action of DNA polymerase and DNA ligase.

It is reported that the other members of MSH2 family function beyond MMR. MSH1 is required for mitochondrial stability (Reenan & Kolodner, 1992) and disruption of MSH1 can influence male sterility in several species (Sandhu, Abdelnoor, & Mackenzie, 2007; Zhao et al., 2016). The MSH4 and MSH5 form heterodimer between them without involving MSH2 and exclusively function in meiosis (Hollingsworth, Ponte, & Halsey, 1995; Lu et al., 2008; Schofield & Hsieh, 2003; Wang et al., 2016). The expression of MSH7 is required for wild‐type level of fertility in barley (Lloyd et al., 2007). MSH7 also plays a role in UV‐B‐induced DNA damage recognition and recombination in Arabidopsis (Lario, Botta, Casati, & Spampinato, 2015).

Among the MMR proteins, MSH2 is a common protein in three of the heterodimers formed in the plants. In conformity of the role of MSH2 protein in repair of replication errors, an Arabidopsis mutant defective in MSH2 shows microsatellite instability (Depeiges, Farget, Degroote, & Picard, 2005). The *msh2* mutants in Moss show strong developmental defects, are sterile, and have a mutator phenotype (Trouiller, Schaefer, Charlot, & Nogue, 2006). A similar phenotype was also observed in *Arabidopsis thaliana msh2* mutant lines albeit only after five generations (Hoffman, Leonard, Lindberg, Bollmann, & Hays, 2004). Considering that suppression of MMR leads to an increase in natural mutation frequency, *MSH2* gene was silenced in *Nicotiana tabacum* and *N. plumbaginifolia* using an artificial microRNA (amiRNA). Although the transformed lines showed developmental and morphological abnormality, the mutant phenotype was not transmitted to subsequent generations (Van Marcke & Angenon, 2013).

Among the interacting partners for MSH2, mutants are reported only for *MSH6* and *MSH7* in Arabidopsis. The *msh6* mutant does not display a visible phenotype (Lario, Ramirez‐Parra, Gutierrez, Casati, & Spampinato, 2011); however, *msh7* mutant shows an increase in meiotic recombination frequency (Lario et al., 2015). The role of *MSH7* in meiotic recombination is also reported in tomato, where RNAi silencing of *MSH7* improves homologous meiotic recombination with a chromosome from *S. lycopersicoides* (Tam, Hays, & Chetelat, 2011). Most likely the high increase in meiotic recombination is mediated by the MSH2‐MSH7 complex as Arabidopsis *msh2* mutant also shows 40% increase in the meiotic crossover rates (Emmanuel, Yehuda, Melamed‐Bessudo, Avivi‐Ragolsky, & Levy, 2006).

In this study, our aim was to isolate MSH2‐defective mutants to obtain a hypermutable line to increase the efficiency of EMS mutagenesis in tomato. We screened natural accessions of tomato and EMS‐mutagenized lines for SNPs/mutation in tomato *MSH2* by EcoTILLING and TILLING, respectively. Considering that little polymorphism was observed in *MSH2* in tomato accessions, we silenced *MSH2* gene using *MSH2‐*RNAi construct. We report that silencing of *MSH2* gene led to a developmental defect in tomato lines with abnormal flowers bearing tetraploid meiocytes in the anthers. The examination of root cells showed the formation of syncytial nuclei and defects in the cell plate formation. Our study indicates that *MSH2* may also have a role in the regulation of meiotic and mitotic cell divisions.

## 2 RESULTS

### 2.1 EcoTILLING of 4‐9 exon of *MSH2* gene showed little polymorphism in tomato accessions

Given the pivotal role of *MSH2* in MMR, we screened a total of 391 tomato accessions for polymorphism in the gene. A 2.8‐Kb region (4–9 exon) of *MSH2* predicted to be deleterious by CODDLE software (http://blocks.fhcrc.org/proweb/coddle/) was chosen for EcoTILLING. Consistent with the essential nature of the gene, only seven accessions showed polymorphism with a total of 13 SNPs with a frequency of 1.23 SNP/100 Kb (Figure S1). Among the identified SNPs, six were nonsynonymous and seven were synonymous (Table 1). The likelihood of nonsynonymous SNPs affecting protein function was examined by SIFT software. As none of the SNP showed a SIFT score <0.05, these SNPs were predicted to be nondeleterious. An in silico analysis of *MSH2* gene in genomes of 54 tomato accessions sequenced by Aflitos et al. (2014) revealed SNPs only in 10 accessions (Table S1). A parallel screening of 1243‐bp region of *MSH2* gene covering 4th exon of 11304 tomato EMS‐mutagenized lines by TILLING did not yield any mutation.

**Table 1:** List of SNPs detected in *MSH2* gene in different tomato accessions along with amino acid changes. The scores predicted by SIFT suggest that none of the amino acid changes would affect MSH2 protein activity

Accession No.	No. of SNPs	Base change	Amino acid change	SIFT Score	Nature of SNP
EC398695	2	G1168T	A77A	–	Synonymous
		T1365C	V143A	1.00	Nonsynonymous
EC520075	3	A1720T	A261A	–	Synonymous
		T1876G	A313A	–	Synonymous
		G3193T	V639V	–	Synonymous
EC520052	1	T1876G	A313A	–	Synonymous
EC521078	4	C1312T	F125F	–	Synonymous
		G1545T	G203V	0.23	Nonsynonymous
		G1808A	D291N	0.83	Nonsynonymous
		G2733T	G545V	–	Nonsynonymous
EC528374	3	G1168T	A77A	–	Synonymous
		T1365C	V143A	1.00	Nonsynonymous
		T1876G	A313A	–	Synonymous
EC34480	4	C1312T	F125F	–	Synonymous
		G1381C	A148A	–	Synonymous
		G1808A	D291N	0.83	Nonsynonymous
		G3777T	A707V	–	Nonsynonymous
EC520078	4	C1312T	F125F	–	Synonymous
		T1339C	A134A	–	Synonymous
		C1796G	Q287E	0.37	Nonsynonymous
		G1808A	D291N	0.83	Nonsynonymous

A SIFT score of less than 0.05 predicts the base substitution to be intolerant.

### 2.2 Generation and molecular analysis of *MSH2*‐RNAi lines

Considering recalcitrance to the mutagenesis and low polymorphism in *MSH2* gene, we silenced the gene by RNAi. A 301‐bp region of *MSH2* gene with no significant homology to any other tomato gene including other MSH genes was amplified and cloned into pHELLSGATE 12 vector (Figure S2). A total of ten independent transgenic lines bearing *MSH2‐*RNAi construct were raised using *Agrobacterium‐*mediated transformation. Southern blot analysis of T_0_ lines revealed the presence of the transgene in five lines (Figure S3). In T_2_ generation out of three lines, the lines 1T_2_1‐11 and 2T_2_7‐5 with strong MSH2 silencing showed a single transgene copy (Figure S4).

**Figure 1:**
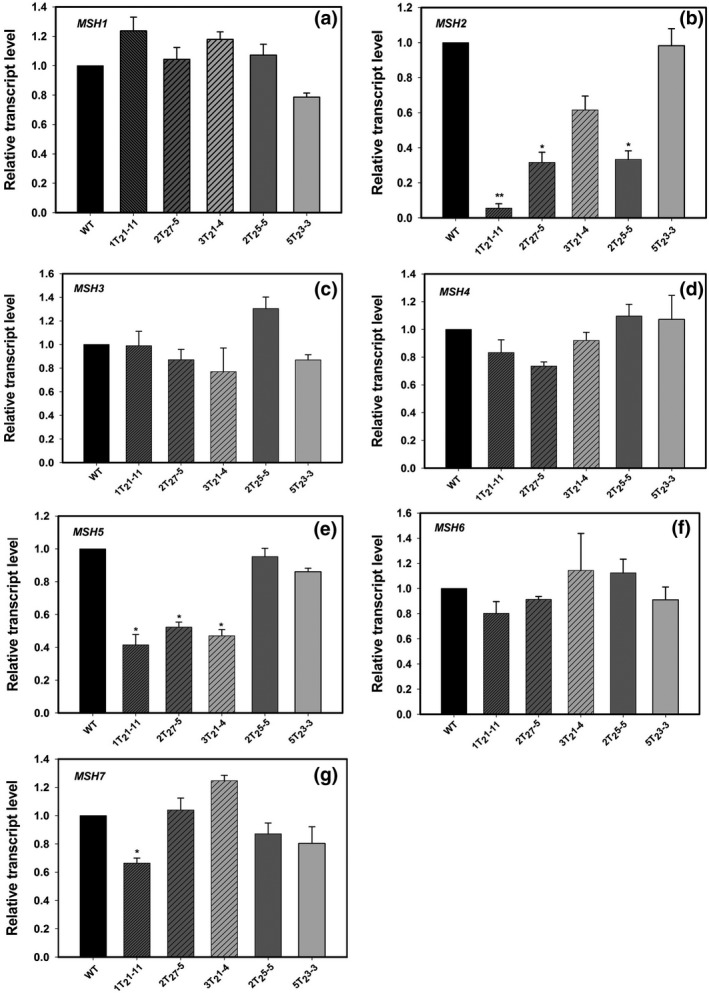
Relative transcript levels of members of *MSH* gene family in leaves of five different *MSH2‐* RNAi lines. (a) *MSH1*, (b) *MSH2*, (c) *MSH3*, (d) *MSH4*, (e) *MSH5*, (f) *MSH6*, (g) *MSH7*. The transcript levels were expressed after normalization with two internal controls, β*‐actin* and *ubiquitin*. Note: line No. 1T_2_1‐11 shows the reduction in *MSH2, MSH5,* and *MSH7* transcript. The statistical significance was determined by Student's *t* test. *p < .05

To ascertain the magnitude of gene silencing, the level of *MSH2* transcript in transgenic lines was quantified by real‐time PCR. Of five Southern positive lines, the line 1T_2_1‐11 showed ~80% reduction in *MSH2* transcript level followed by lines 2T_2_5‐5 and 2T_2_7‐5 compared to wild‐type (WT) control (Figure 1). Line 3T_2_1‐4 showed moderate and 5T_2_3‐3 showed no reduction in *MSH2* transcript levels. The reduction in the transcript level in line 1T_2_1‐11 was not restricted to *MSH2* alone; it also reduced the transcript levels of *MSH5* and *MSH7* albeit to a moderate but a significant extent. As *MSH2*,* MSH5,* and *MSH7* genes bear no significant sequence homology, the reduction in *MSH5* and *MSH7* transcript is most probably due to some indirect influence of *MSH2* on this transcript reduction.

To locate the site of transgene insertion, FPNI‐PCR was performed on genomic DNA of 1T_2_1‐11 and 2T_2_7‐5 tetraploid *MSH2*‐RNAi lines that showed strong reduction in the transcript of the *MSH2* gene. The amplified PCR product flanking the right border of inserted T‐DNA was cloned and sequenced. The flanking sequence of line 1T_2_1‐11 showed 98% homology to *Tic22* gene in chromosome No. 9 (Table S2, Figure S5a,b) and that of 2T_2_7‐5 showed hit in *TRM32* gene, a member of the TON1 Recruiting Motif superfamily (Table S2, Figure S5c,d). While TRM1 reportedly associates with cortical microtubules, not all TRM proteins associate with microtubules. Moreover, the sequence diversity among the TRM family members with varied transcriptional expression patterns indicates that different members may have different roles (Struk & Dhonukshe, 2014).

### 2.3 *MSH2* silencing exacerbates UV‐B‐mediated thymine dimer formation

**Figure 2:**
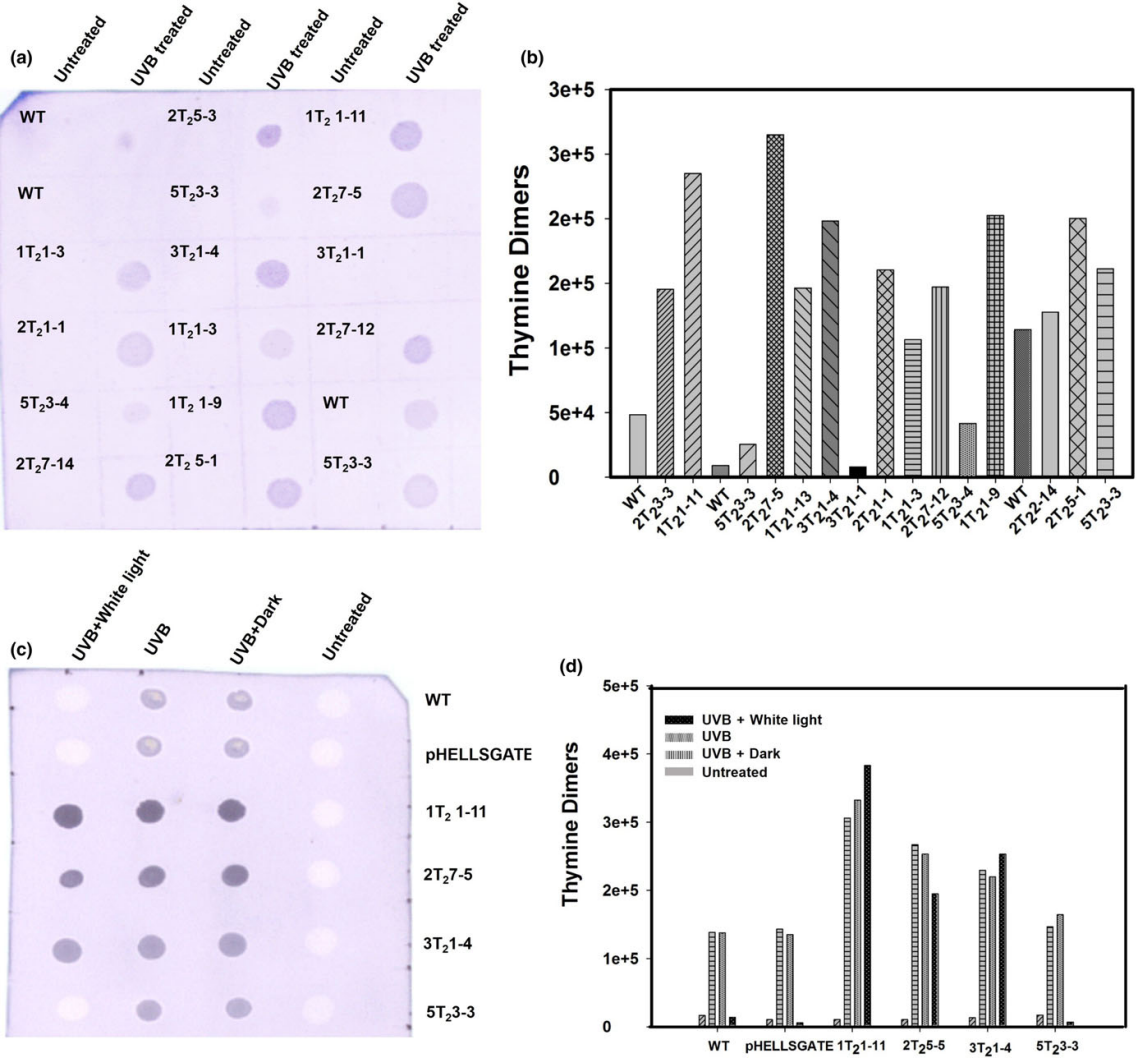
Determination of the in vivo activity of MSH2 by quantification of thymine dimer levels using a thymine dimer‐specific antibody in UV‐B‐irradiated WT and *MSH2‐* RNAi lines by dot‐blot assay. (a, b) Quantification of thymine dimers in WT and *MSH2‐* RNAi plants after 6 hr with or without UV‐B exposure (untreated). (a) Representative dot blot. The label on top of each spot indicates the line number of *MSH2‐*silenced line. (b) Quantification of thymine dimers in WT and *MSH2‐* RNAi lines by ImageJ analysis. (c, d) Quantification of thymine dimers in WT and *MSH2‐* RNAi plants. Thymine dimers were quantified either immediately after 6‐hr exposure with UV‐B, or after 24 hr of white light, or dark incubation. (c) Representative dot blot. The labels on the right of the blot indicate the line number of *MSH2‐*silenced line. (d) Quantification of thymine dimers in *MSH2‐* RNAi lines by ImageJ analysis. Note: The thymine dimers in (b) and (d) were quantified using three independent biological replicates. The statistical significance was determined by Student's *t* test. *p < .05 [Color figure can be viewed at wileyonlinelibrary.com]

In mammalian cells, the contribution of the MMR pathway to the UV‐B‐induced DNA damage is well established (Narine, Felton, Parker, Tron, & Andrew, 2007; Seifert et al., 2008). Similarly, Arabidopsis *MSH2* T‐DNA insertion lines show higher levels of cyclobutane pyrimidine dimer (CPD) on exposure to UV‐B (Lario et al., 2011, 2015). We used UV‐B‐induced thymine dimer formation as an indirect assay to monitor the activity of MSH2 in transgenic plants in comparison with WT. Pre‐ and post‐UV‐B exposure, the thymine dimers were quantified in genomic DNA of above plants using a thymine dimer‐specific monoclonal antibody (Figure S6, Figure 2). Prior to UV‐B exposure, the steady‐state levels of thymine dimers in control and *MSH2*‐silenced lines were nearly similar. However, after 6‐hr UV‐B exposure, *MSH2*‐silenced lines showed a high level of dimers than the control plants. The thymine dimer level in *MSH2‐*RNAi lines was higher than the control even after 24‐hr dark or white light incubation (Figure 2). It appears that high accumulation of thymine dimer in M*SH2* transgenic lines may have resulted from low in vivo MMR activity.

### 2.4 *MSH2*‐RNAi lines display abnormal pollen formation

**Figure 3:**
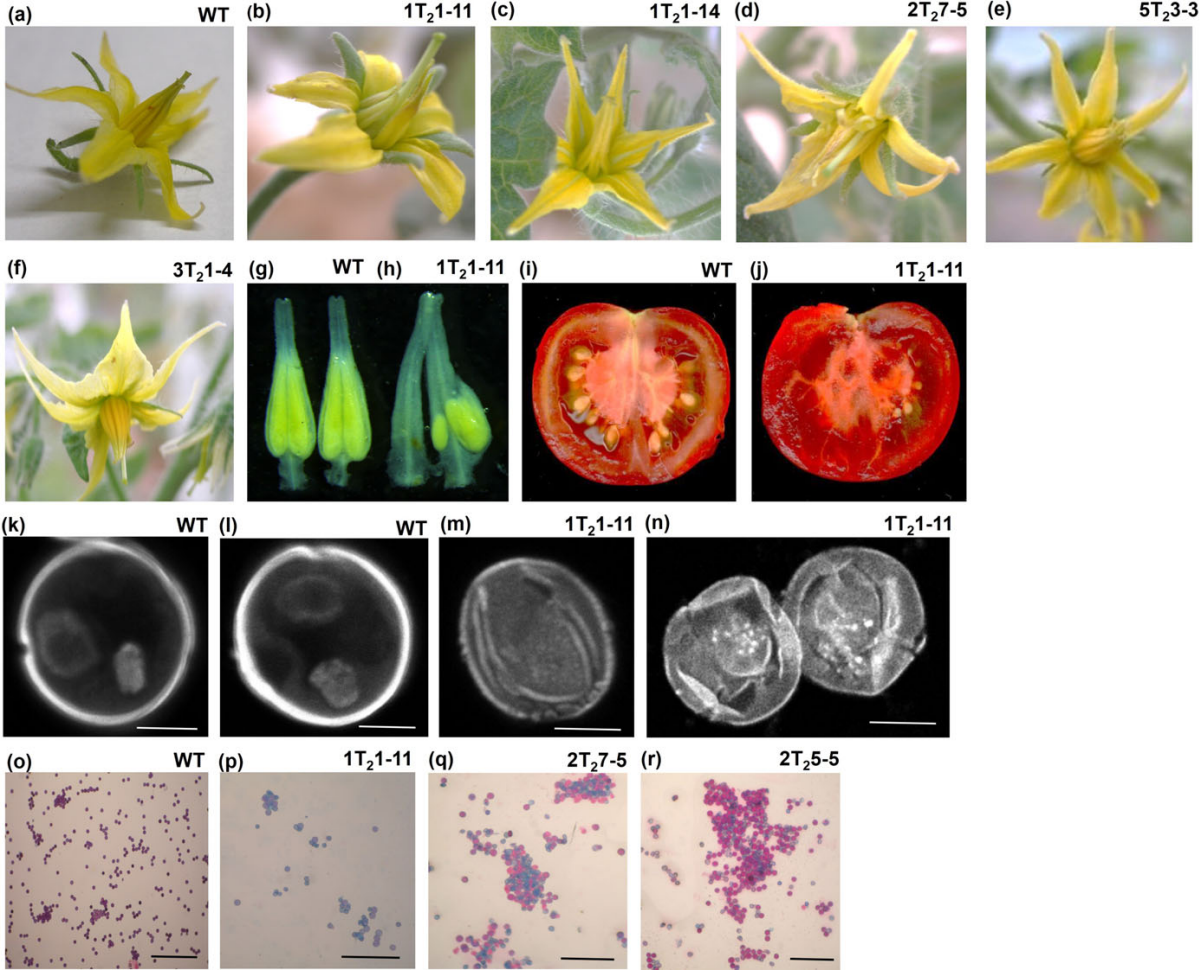
Abnormal flower, anther, fruit, and pollen morphology manifested by *MSH2‐* RNAi T_2_ plants. (a) WT flower (b–d) Abnormal floral morphology, particularly in stamens, in strongly *MSH2‐*silenced lines. (e–f) Flowers of partially *MSH2*‐silenced lines, 5T_2_3‐3 and 3T_2_1‐4, are nearly similar to the WT. (g) Anther cones of line WT. (h) Abnormal anther cones of line 1T_2_1‐11. Note the absence of characteristic yellow coloration in 1T_2_1‐11 anther cone on left and partial coloration near the base of anther cone on right. (i–j) Vertical cut section of red ripe fruit showing highly reduced seed set in line 1T_2_1‐11 (i) compared to WT (j). (k–l) WT pollen grains are bicellular with vegetative and generative cells compared to empty (m) and shriveled (n) pollen grain in line 1T_2_1‐11 (K‐N, Scale bar, 20 μm). (o–r) Alexander staining showing viable pollen grains in WT (o), lines 1T_2_1‐11 (p), 2T_2_7‐5 (q), and 2T_2_5‐5 (r). (l‐o, Scale bar, 200 μm)

The *MSH2‐*RNAi lines showed slower growth and displayed mild phenotypic differences compared to nontransgenic lines during vegetative growth (Figure S7). In contrast, the lines 1T_2_1‐11 and 2T_2_7‐5 with a higher reduction in *MSH2* transcript also showed floral abnormalities in T_1_ and T_2_ generation (Figure 3a–f). The most pronounced abnormality was in the stamen morphology (Figure 3g–h). The plants in later generations too showed abnormalities in flowers, mostly in the stamens. The progeny plants of silenced lines either failed to set fruits or set fruits with very few seeds (Figure 3i–j). These seeds on germination showed a variable degree of viability. The viable seeds albeit in reduced number were propagated to the next generation.

The examination of pollens revealed that strongly silenced lines produced pollens with the deformed shape. Unlike WT pollens that were round with a vegetative and generative cell (Figure 3k–l), the pollens of silenced lines showed the deformed shape and were either empty or contained the remnant of chromosome fragments (Figure 3m–n). It is likely that pollen grains collapsed either due to lack of nourishment in deformed anthers or *MSH2* silencing affected some other gene.

The viability of WT and *MSH2‐*RNAi pollens was examined by Alexander staining. In WT, all pollens exhibited a red‐purple fluorescence, strongly reflecting the high male gametophytic fertility of these plants (Figure 3o). In contrast, *MSH2‐*RNAi lines such as 1T_2_1‐11 displayed mostly inviable pollens (Figure 3p–r). As a consequence, *MSH2‐*RNAi plants set fruits bearing ca. 5% seeds. The tomato line 1T_2_1‐11 with the highest reduction in *MSH2* expression showed a maximum loss in the fertility with >90% blue‐stained pollen grains (Figure 3p). The fruits of 1T_2_1‐11 line bore very few viable seeds along with underdeveloped seeds (Figure 3j).

### 2.5 *MSH2*‐RNAi lines display tetraploid meiosis progression

**Figure 4:**
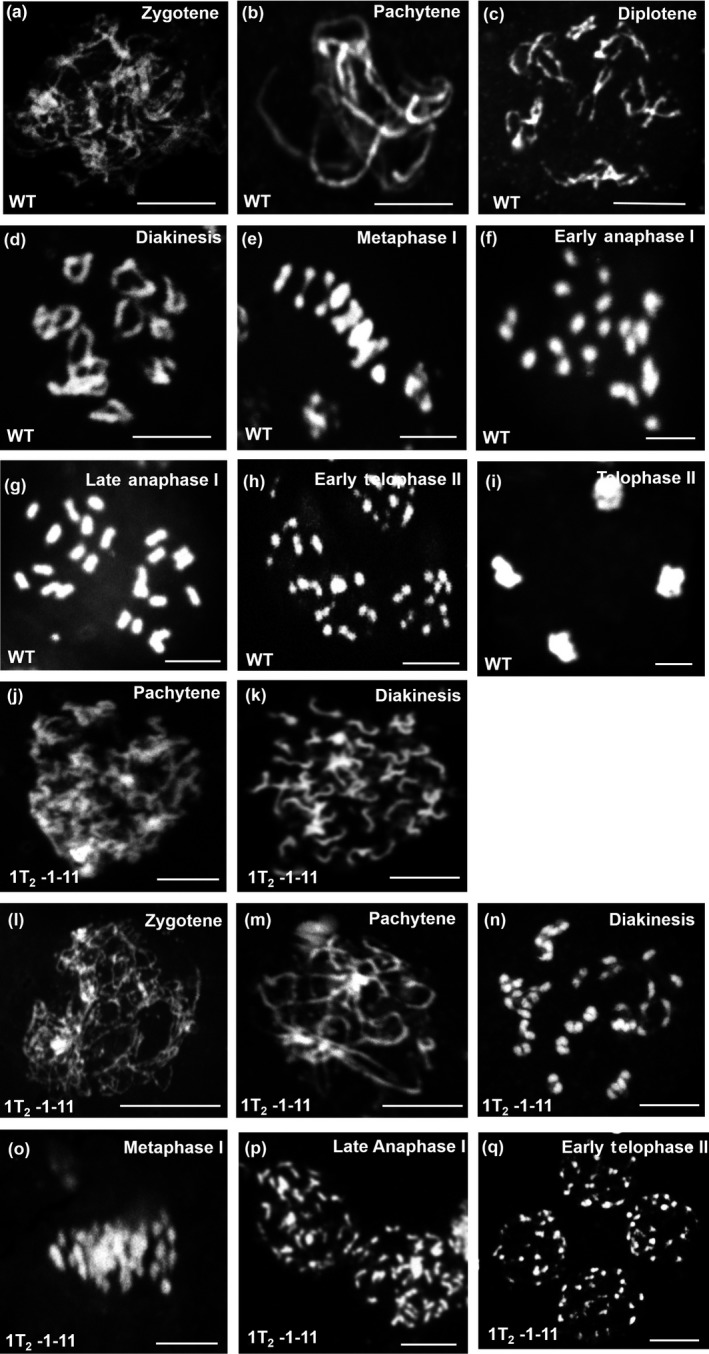
Male meiosis in wild‐type and *MSH2‐* RNAi tomato lines. Representative meiotic stages of wild‐type (a–i) and *MSH2‐* RNAi (j–q) lines from zygotene to telophase. In *MSH2‐* RNAi meiocytes, few univalent chromosomes were observed that were assumed to be in early prophase Ι (j–k). A proportion of male meiocytes of *MSH2‐* RNAi lines underwent tetraploid meiosis that progressed from prophase to telophase (l–q). Note the formation of diploid tetrads (q). Scale bar, 10 μm

The deformities in the anther morphology are reported to be associated with defects in male meiosis in tomato (Jeong et al., 2014; Rick, 1948) and Arabidopsis (Panoli et al., 2006). Considering that observed stamen abnormalities could be associated with meiosis, we examined meiosis progression in the *MSH2*‐silenced lines. The pollen mother cells (PMCs) were isolated at different stages of floral bud development as outlined by Brukhin, Hernould, Gonzalez, Chevalier, and Mouras (2003) from *MSH2*‐silenced lines and WT. A complete meiotic progression was examined for WT which progressed normally with characteristic landmarks for different stages of the meiosis, such as zygotene (Figure 4a), pachytene (Figure 4b), diplotene and diakinesis (Figure 4c–d) followed by metaphase (Figure 4e), early and late anaphase (Figure 4f–g), early and late telophase (Figure 4h–i).

In strongly silenced *MSH2‐*RNAi lines, irrespective of the stage of floral bud development, predominant short condensed chromosomes were observed in the majority of the meiocytes. These meiocytes were either arrested at the prophase I (Figure 4j–k) or completed later stages of meiosis (Figure 4l–q). The abnormal increase in the number of chromosomes in meiocytes was suggestive of either premature loss of sister chromatid cohesion or increase in ploidy leading to the formation of diploid tetrads (Figure 4q). In these lines, very few meiocytes were observed with WT complement of chromosomes.

**Figure 5:**
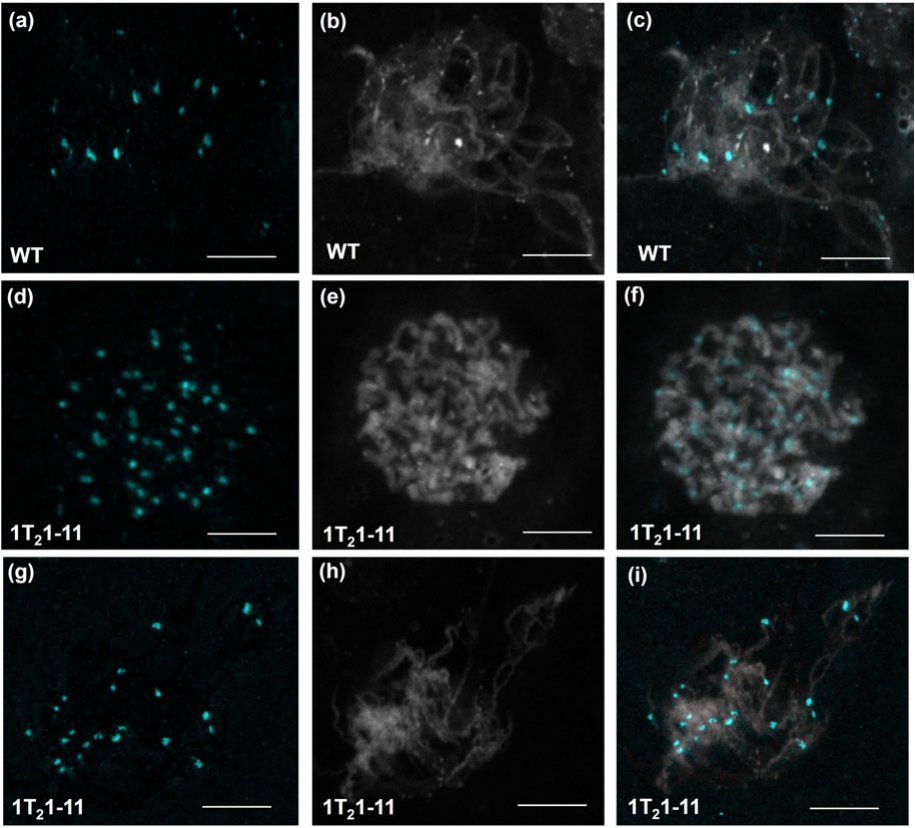
Immunolocalization of centromere marker protein—CENPC—in nuclei of WT and *MSH2‐* RNAi, 1T_2_1‐11 line during prophase I. For each nucleus, the CENPC and DAPI staining along with merged signals is shown. (a–c) Zygotene nuclei with 12 centromere spots are likely indicating 12 bivalents in WT (a) with DAPI (b) and merged image (c). (d–f) Assumed zygotene nuclei with 48 centromere spots in 1T_2_1‐11 line (d) are likely indicating 48 univalents along with DAPI (e) and merged image (f). (g–i) Assumed zygotene nuclei with 24 centromere spots in line 1T_2_1‐11 (g) likely indicating the presence of 24 bivalents along with DAPI (h) and merged images (i). The nuclei were examined using a 63× (1.4 N.A.) oil‐immersion objective on a Leica SP5 confocal microscope. Scale Bar, 10 μm

To quantify the chromosome number in meiotic cells of *MSH2‐*RNAi lines, we used centromere protein C (CENPC) antibody that specifically labels the centromere. Immunolabeling of WT prophase Ι meiocytes with CENPC antibody showed 12 centromeres at pachytene, indicating the likely presence of 12 bivalent chromosomes (Figure 5a–c). In contrast, the meiocytes of *MSH2*‐silenced line at a stage equivalent to prophase Ι showed 48 centromeres (Figure 5d–f). There are two possibilities to explain the presence of 48 centromeres. First, a loss of sister chromatid cohesion in the prophase I may lead to the appearance of 48 sister chromatids. Second, an increase in ploidy to tetraploid in the pollen mother cell followed by an absence or delay in sister chromatid cohesion may result in 48 chromosomes. Analysis of meiotic stages favors the second scenario where we can observe the occurrence of diploid tetrads where the sister chromatids separate during anaphase II. Consistent with above, among the meiocytes examined, most contained 48 chromosomes as signified by CENPC; however, in few meiocytes, the meiosis proceeded to anaphase Ι with segregation of 24 chromosomes to each pole (Figure 5g–i). Beyond this, other stages of meiosis I and meiosis II were rarely observed indicating a block in the meiosis of the silenced lines. However, a few cells displayed 12 centromeres, indicating that silenced lines generate haploid spores albeit at highly reduced level.

We observed that only a few tetraploid cells completed meiosis II with the formation of diploid tetrads at the end of meiosis II (Figure 4k–o). The analysis of relative proportions of meiocytes revealed that in 1T_2_1‐11 line, 25% meiocytes displayed abnormal tetraploid chromosomes, 67% displayed tetraploid meiocytes, and 8% displayed diploid meiosis (Table 2). Similarly, the 2T_2_7‐5 line had 25% meiocytes with abnormal tetraploid and 75% tetraploid nuclei giving rise to diploid microspores. Consistent with this, the anthers of *MSH2*‐silenced lines contained largely empty shrunken pollen at the end of meiosis. Taken together, these results confirm that *MSH2‐*RNAi meiocytes display abnormal meiotic progression resulting in tetraploid meiocytes. The tetraploid meiocytes were observed only in 1T_2_1‐11 and 2T_2_7‐5 lines with high silencing of *MSH2* transcript. The 3T_2_1‐4 line with moderate *MSH2* silencing and 5T_2_3‐3 line with no *MSH2* silencing displayed diploid meiocytes similar to WT (Figure S8).

**Table 2:** Number of male meiocytes in different *MSH2‐*RNAi lines with reference to WT and the number of seeds per fruit in the respective lines

Line No	Total No. of meiocytes count	No. of Diploid meiocytes	No. of Normal Tetraploid meiocytes	No. of Abnormal Tetraploid meiocytes	No. of seeds per fruit
WT	60	60	0	0	40–50
1T_2_1‐11	60	5	40	15	0–5
2T_2_7‐5	60	0	45	15	2–10
3T_2_1‐4	60	60	0	0	40–50
5T_2_3‐3	60	60	0	0	40–50

### 2.6 Root tip cells show diploid nuclei with sporadic defect in cell plate formation

In this study, *MSH2‐*RNAi tomato lines generated tetraploid meiocytes. However, no predominant enlarged organ size, a characteristic of increased ploidy, was observed during the vegetative growth of plants (Figure S7). Even though the silenced lines displayed abnormal floral organs, these too showed no significant enlargement of floral structures. To ascertain whether *MSH2* silencing leads to tetraploidy in the progeny of the plants, we examined nuclear volumes in root tips of silenced and control seedlings after staining of nuclei with DAPI (Figure S9). Interestingly, the *MSH2‐*RNAi lines 1T_2_1‐11 and 2T_2_7‐5 with tetraploid meiocytes also displayed nuclear volumes similar to WT and empty vector lines, indicating that these plants were diploid (Bourdon et al., 2012). Considering that the DAPI staining of root tip nuclei of tetraploid LA3255 line showed bigger nuclear volumes than diploid wild‐type and *MSH2‐*RNAi lines (Figure S9), it affirms that the root tips of seedlings of strongly silenced *MSH2‐*RNAi lines were diploid.

**Figure 6:**
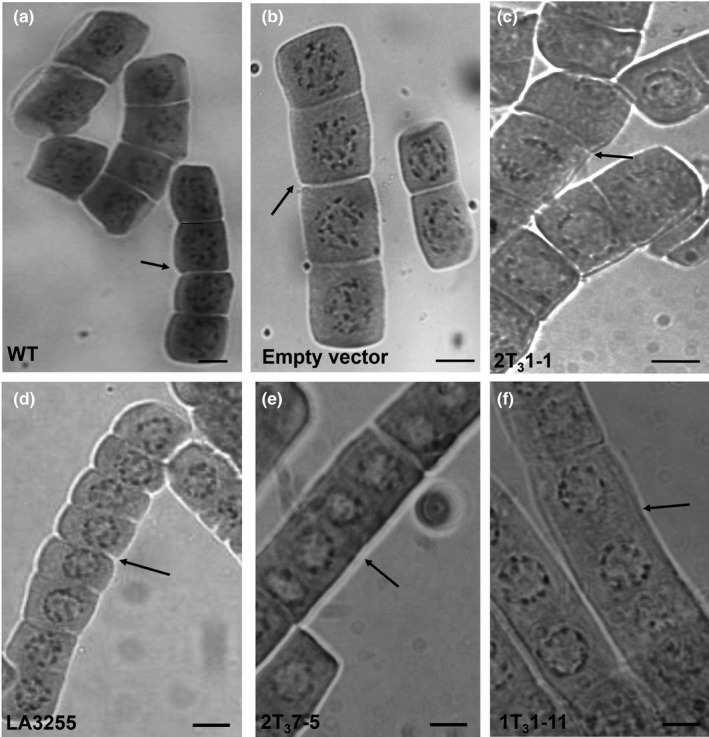
Mitosis in WT and T_3_ *MSH2‐* RNAi lines root tip cells of progenies of (a‐d). WT (a), empty vector control lines (b), Progenies of *MSH2‐* RNAi*,* 2T_2_1‐1 line bearing diploid meiocytes (c), and tetraploid line LA3255 (d) showing clear separation of the diploid nucleus in mitotic cells of root tip by intervening cell plate. (e–f). Progenies of strongly silenced *MSH2‐* RNAi lines 2T_2_5‐1 (e) and 1T_2_1‐11 (f) with tetraploid meiocytes show the absence of cell plate between dividing mitotic nuclei. Scale Bar, 100 μm

At the same time, few cells showed no intervening cell wall between the nuclei, suggesting that *MSH2* silencing affected the cell plate formation (Figure 6). The loss of intervening cell wall resulted in the formation of several syncytial nuclei within a cell (Figure 6e,f). In contrast, the weakly silenced *MSH2‐*RNAi lines with diploid meiocytes exhibited root tip with uniformly sized diploid nuclei with distinct cell plate formation (Figure 6c), indicating that loss of cell plate was a feature specific to lines with a higher degree of *MSH2* silencing. To confirm whether loss of cell plate formation was related to the tetraploidy, we examined root tips of tomato tetraploid line, LA3255. The root cells of the LA3255 line showed normal tetraploid nuclei with no defect in the cell plate formation (Figure 6d).

## 3 DISCUSSION

The DNA mismatch repair is the highly conserved biological pathway that maintains the fidelity of DNA replication and genetic stability. In the mammalian system, the defects in MMR lead to abnormalities in meiosis and increase predisposition to certain types of cancer (Li, 2008). Consistent with the vital role of MMR, the *MSH2* in tomato appeared recalcitrant to mutagenesis, as evident by the absence of nonsynonymous mutations in M2 lines screened by TILLING. In the mammalian system, the polymorphism in *MSH2* significantly increases the risk of breast cancer (Hsieh et al., 2016). The paucity of polymorphism in *MSH2* as revealed by EcoTILLING of tomato accessions is in conformity with the indispensable nature of the gene. Although a missense and few silent mutations were identified, these did not influence the protein function as predicted by SIFT.

In plants, the role of *MSH2* gene in MMR has been mainly inferred based on its homology with yeast and mammalian genes. In Arabidopsis, a *MSH2* T‐DNA insertional mutation accumulates mutations in microsatellites and other loci indicating a role of *MSH2* in MMR (Hoffman et al., 2004; Leonard, Bollmann, & Hays, 2003). MSH2 reportedly plays a role in repair of the UV‐B‐induced DNA damage in the mammalian system (Peters, Young, Maeda, Tron, & Andrew, 2003; Seifert et al., 2008). In plants as well, MSH2 likely plays a role in the repair of UV‐B‐induced cyclobutane pyrimidine dimer (CPD). The Arabidopsis *MSH2* mutant on UV‐B exposure shows enhanced formation of CPD suggesting a role of MSH2 in the repair of UV‐B damage (Lario et al., 2011). Similarly, in strongly silenced tomato *MSH2‐*RNAi lines, thymine dimer formation is exacerbated by exposure to UV‐B. Also, *MSH2‐*deficient plants show inefficient repair of thymine dimers even on exposure to visible light that stimulates photoreactivation repair. From the preceding, it is apparent that strongly silenced *MSH2‐*RNAi lines have low in vivo MMR activity as evident by enhanced UV‐B damage.

The propagation of Arabidopsis *MSH2* T‐DNA insertional mutant line revealed the accumulation of a wide variety of mutations such as morphological abnormalities, reduction in seed/silique development, and loss of germination efficiency (Hoffman et al., 2004). The strongly silenced tomato *MSH2‐*RNAi lines show mild abnormalities during vegetative development, but showed defects during floral development with abnormal anthers. The lines with abnormal stamens showed reduced fruit set and highly reduced seed numbers in the fruits. In contrast to Arabidopsis *MSH2* mutant lines where seed set on average was reduced to only 50% (Hoffman et al., 2004), in tomato silenced lines, it was less than 10%.

The reduction in seed set can result from the failure in the embryo development. Considering that the *MSH2*‐silenced lines developed abnormal anthers, reduced seed set may have resulted from the failure to make viable pollens in the deformed anthers. Consistent with this, Alexander staining showed nonviable pollens with blue staining in highly silenced lines. As WT and moderately silenced lines showed viable pollens with red staining, it can be assumed that *MSH2* silencing leads to loss of pollen viability in tomato. Thus, the reduced seed set in the silenced lines is in consonance with the loss of pollen viability.

The successful pollen development in plants requires operation of proper meiosis in the developing anther (Ma, 2005). In several plants, defects in the anther formation are associated with defective meiosis or meiotic arrest (Walbot & Egger, 2016). In tomato, heat‐induced abnormal anther development in a conditional male sterile mutant is associated with reduced pollen set and loss of pollen viability (Rick & Boynton, 1967). It is, therefore, likely that silencing of MSH2 expression affected anther development that in turn may have affected the progression of meiosis.

Consistent with this view, the *MSH2*‐silenced lines showed a meiotic arrest in the anthers. The majority of pollen mother cells (PMC) of *MSH2‐*RNAi lines fail to complete the meiotic cycle. These PMCs initiate meiosis as evident by the onset of zygotene. However, the meiosis gets stalled at the final stage of prophase I—diakinesis, as the chromosomes are at their most condensed form. The stalling at diakinesis is seemingly related to the increase in the ploidy as CENPC‐stained cells showed the presence of 48 univalent chromosomes. The stalling of the meiosis is not total, as few CENPC‐stained cells also showed 24 bivalent chromosomes, which completed meiosis presumably generating diploid pollens. However, most pollens were shrunken, and only a few showed Alexander staining. It is plausible that loss of pollen viability was largely for the diploid pollens. From the preceding, it is likely that incomplete meiosis and the formation of nonviable pollens contribute to the reduced seed set in the strongly silenced *MSH2‐*RNAi lines.

The process that generates tetraploid nuclei in the PMC remains to be investigated. In several plants including tomato during microsporogenesis, the nuclei can transfer to the neighboring cell by cytomixis (Mursalimov, Sidorchuk, & Deineko, 2013; Weiling, 1965). Such intercellular transfer of nuclei can influence the ploidy level of the produced pollens. However, such cytomictic transfer of nuclei most probably does not contribute to tetraploid nuclei, as in tomato cytomixis is observed only ca. 15% of PMC (Weiling, 1965). The occurrence of a large number of polyploid pollen mother cells does not favor cytomixis to be the cause of tetraploid nuclei. The probability that tetraploid meiocytes arise due to *MSH2* silencing‐induced endomitosis also seems remote. In Chrysanthemum, the fusion of two PMC forms tetraploids, albeit this process occurs at very low frequency (Kim, Oginuma, & Tobe, 2009), and cannot account for high numbers of tetraploid meiocytes in *MSH2*‐silenced lines.

The root cells of *MSH2‐*RNAi‐silenced lines show defective cytokinesis with multiple nuclei in syncytial cells. The similar syncytial cells may also form due to defective cytokinesis during meiocytes archesporial cell development. It is likely that the fusion of nuclei in syncytial cells gives rise to the tetraploid nuclei in PMC of *MSH2‐*RNAi‐silenced lines. The fusion of nuclei resulting in the stochastic formation of tetraploid PMC initials has been documented in mutants defective in cell plate formation. In Arabidopsis, the mutations in sterol methyl transferase and callose synthase lead to defect in cell plate formation predominantly in the floral tissues. Both mutants also form tetraploid meiocytes and diploid gametes in both male and female sporogenesis (De Storme et al., 2013). The formation of tetraploid meiocytes in *MSH2*‐silenced lines is analogous to tomato *pmcd1* mutant, where cytokinesis failure in meiocytes archesporial cells generates tetraploid nuclei (De Storme & Geelen, 2013a).

The *MSH2*‐silenced lines show the formation of the very few viable pollens and the progeny of these plants was exclusively diploid. The strongly reduced seed set in silenced lines seems to indicate that only diploid embryos survived postfertilization. Another reason could be defective endosperm development due to unbalanced 1:1 ratio rather than normal 1:2 ratio of the paternal and maternal genome (Scott, Spielman, Bailey, & Dickinson, 1998). The absence of triploid progeny may also result from the existence of strong triploid block in members of Solanaceae including tomato (Ehlenfeldt & Ortiz, 1995; Nilsson, 1950). The absence of triploid or tetraploid progeny in *MSH2*‐RNAi lines is also consistent with the reported absence of triploid/tetraploid plants in progeny of tomato *pmcd1* mutant that shows a cytokinesis defect and generates diploid pollens (De Storme & Geelen, 2013a).

The premeiotic doubling of chromosome occurs in unisexual and parthenogenetically reproducing animal species (Itono et al., 2006). It is believed that in higher plants, such chromosomal doubling rarely occurs (De Storme & Geelen, 2013b). In higher plants, the doubling of chromosomes reportedly occurs after the onset of meiosis via meiotic restitution (De Storme & Geelen, 2013b). Considering that doubling of chromosomes in *MSH2*‐silenced line occurs before the onset of meiosis, it is of a novel nature. The very high frequency of tetraploid meiocytes also hints for a likely function of *MSH2* in the regulation of chromosomal doubling via a mechanism that remains to be deciphered. As the formation of tetraploid meiocytes in *MSH2*‐silenced line is quite similar to *pmcd1* mutant, the failure of cytokinesis appears to be the main contributor to this phenomenon.

In plants, MSH2 heterodimerizes with MSH3, MSH6, and MSH7. However, none of these heterodimers reportedly have any function in the regulation of meiosis. Conversely, the *MSH4* and *MSH5* mutants in Arabidopsis and rice though exhibit normal vegetative growth and show severe fertility reduction likely related to meiotic defects (Higgins, Armstrong, Franklin, & Jones, 2004; Lu et al., 2008; Luo et al., 2013; Wang et al., 2016). The *MSH2*‐silenced lines also affected *MSH5* and *MSH7* transcript levels which may have affected the process of meiosis. The manifested tetraploid meiocyte formation and later failure in meiosis might be due to the reduction of these three transcripts. Alternately, the increased microsatellite instability caused due to low activity of the MSH2 protein may contribute to defective cytokinesis and tetraploid meiocyte formation.

Similar to *MSH2* silencing, loss‐of‐function lines in 40S ribosomal protein S6 kinases in Arabidopsis generate tetraploid PMCs and show a high degree of pollen abortion (Henriques et al., 2010). Likewise, mutations in glucan synthase‐like 8 and sterol methyl transferase 2 lead to defects in somatic cell plate formation and the occasional fusion of syncytial nuclei (Chen et al., 2009; Guseman et al., 2010). It is, therefore, likely that T‐DNA insertion in a meiosis/cytokinesis‐related gene may have caused the tetraploidy. The examination of two silenced lines indicated insertion in *TIC22* and *TRM32* genes; both of these genes have no reported role in meiosis or cell plate formation. Despite having an insertion in widely unrelated genes, both the silenced lines showed identical phenotype, suggesting a close correlation between *MSH2* silencing and tetraploidy.

The tetraploid formation in *MSH2*‐silenced lines is more akin to the proposal that DNA replication or repair defects might produce signals that block cytokinesis and yield tetraploids in cancer cells (Ganem, Storchova, & Pellman, 2007). While it is not reported in plants, in mice embryo fibroblasts, deficiency of *MSH2* in conjunction with *P53* revealed that MSH2 plays a role in preventing polyploidization of cells (Strathdee, Sansom, Sim, Clarke, & Brown, 2001). In mammalian cells, *MSH2* has been identified as a target of the E2F transcription factors (Ren et al., 2002) wherein the loss of *MSH2* inhibits the interaction of *E2F* with *MSH2*, thus affecting the *E2F* signaling. It is believed that disruption of the *E2F* signaling affects the progression of cell cycle and promotes failure of cytokinesis. As *MSH2* have a probable role in mammalian cytokinesis, it is plausible that *MSH2* may affect cytokinesis in plants in a similar fashion. Nonetheless, it remains to be determined whether *MSH2* alone or with its interacting partners plays a direct or an indirect role in regulation ploidy and cytokinesis in plants. It is also likely that *MSH2* silencing leads to wide ramification in plant cells and observed change in the ploidy level reflects its downstream effect.

In summary, our study indicates that *MSH2* silencing leads to tetraploid meiocyte formation in tomato. Additionally, *MSH2* silencing also affects cytokinesis with multiple nuclei in syncytial cells. Although the precise mechanism of *MSH2* action remains to be deciphered, these phenotypes indicate an additional role for *MSH* gene family. Our results also suggest that similar to cytomixis, the formation of syncytial cells may contribute to the tetraploid formation in higher plants. Uncovering the role of MSH2 in the regulation of cytokinesis and nuclear fusion will be of great interest in future studies and may contribute to an alternate route to the polyploid formation.

## 4 EXPERIMENTAL PROCEDURES

### 4.1 Screening for SNPs/mutations in *MSH2* gene

The population used for the EcoTILLING was same as described earlier in Mohan et al. (2016) and Upadhyaya et al. (2017). Genomic DNA was isolated from tomato accessions or EMS‐mutagenized M_2_ lines following the procedure of Sreelakshmi et al. (2010). *MSH2* gene sequence was obtained from SGN (solgenomics.net, Solyc06g069230). CODDLE software (blocks.fhcrc.org/proweb/coddle) was used to predict the most deleterious region of the gene. The primers for SNP/mutation screening were designed using Primer3web version 4.0.0 (bioinfo.ut.ee/primer3) (Table S3). The SNP/mutation detection and haplotype assignment were carried out as described by Mohan et al. (2016). The sequence variations (substitutions and indels) were detected using “Multalin” software (http://multalin.toulouse.inra.fr/multalin/, Corpet (1988). The SNP frequency was calculated using the formula: (total number of SNPs detected/total length of the screened fragment) × 1000 (Frerichmann et al., 2013). PARSESNP (Taylor & Greene, 2003) was used to analyze and display the variation in gene sequences, and SIFT (sorting intolerant from tolerant; Sim et al., 2012) was used to predict the deleterious variations for protein functions.

### 4.2 Cloning of a fragment of *MSH2* gene in pHELLSGATE 12 RNAi vector

For RNAi silencing, a 301‐bp region in 4th exon of *MSH2* showing no significant homology with any other tomato gene was selected. The 301‐bp region from *MSH2* cDNA was PCR‐amplified using primers set with the attB sequence at the 5ʹ end (FP‐AGATTCTCCAGTGATTGTTGCT; RP‐ CAGATCCTGTACCAAATCCCTA). The PCR product was cloned into a pDONR201 vector with attP1 and attP2 sites using BP Clonase enzyme (*Invitrogen*). The gene fragment from the intermediate clone was transferred to the pHELLSGATE12 vector (CSIRO, Australia) by recombination between attL1/attL2 and attR1/attR2, mediated by LR Clonase enzyme (*Invitrogen*). The confirmed clone was transformed into *Agrobacterium tumefaciens* C58C1 strain.

### 4.3 Generation of *MSH2*‐RNAi lines

Tomato (cv. Arka Vikas) transformation was performed as described earlier (Sharma, Solanke, Jani, Singh, & Sharma, 2009) yielding ten independent transgenic lines. The phenotyping of the transgenic plants was continually carried out in successive generations. The lines with 80%–90% *MSH2* transcript silencing showed anther abnormalities. Few T_2_ lines with very strong anther defects did not survive as they produced insufficient viable pollens and failed to set fruits. Therefore, the flowers of the lines with anther abnormalities and drastic reduction in MSH2 transcript levels were manually pollinated with wild‐type pollens to propagate to next generation. The plants were carried forward till T_4_ generation. The following nomenclature was adopted for progressive generations; line No. 2T_0_ indicates T_0_ line No. 2, 2T_1_5 indicates T_1_ progeny No. 5 of line No. 2, 2T_2_5‐7 indicates its T_2_ progeny No. 7 of T_1_ plant No. 5 of T_0_ line No. 2.

### 4.4 Southern blot analysis

Genomic DNA (~15 μg) of T_0_ transgenic lines was digested with BamHI and SalI to release a fragment size of 2.2 Kb (Figure S3), while genomic DNA (~15 μg) of T_2_ transgenic lines was digested EcoRΙ or NotΙ or PacΙ (Figure S4) at 37°C overnight, separated on a 0.8% (w/v) agarose gel, and transferred to Hybond N^+^ membranes. αP^32^‐dCTP‐labeled 2.2‐Kb BamHI‐ and SalI‐digested (Figure S3) and *NPT*ΙΙ (Figure S4) fragments were synthesized by PCR and used as the probe. The primers for amplification of *NPT*ΙΙ probe amplify a 554‐bp fragment in the coding region of *NPT*ΙΙ. Prehybridization, hybridization, washing, and developing of the blot were performed according to Sambrook and Russell (2001).

### 4.5 RNA isolation and quantitative real‐time PCR

RNA was isolated from young leaves with three independent replicates using the method as described earlier (Gupta et al., 2014). Complementary DNA (cDNA) was prepared from 2 μg of RNA using high‐capacity cDNA Archive kit (Applied Biosystems, USA). Quantitative real‐time PCR was also performed as described earlier by Gupta et al. (2014). Gene‐specific primers were designed from the sequences obtained from the SGN database using Primer 3 software (Table S4) while real‐time data analysis was same as described by Gupta et al. (2014).

### 4.6 Analysis of in vivo MSH2 activity

The in vivo MSH2 activity was determined by measuring the amount of thymine dimer formation on exposure to UV‐B light as described by Lario et al. (2011, 2015). Three samples of juvenile 6th to 7th node leaves were exposed for 6 hr to UV‐B radiation (2 Wm^−2^, TL 20 W/01‐RS, Philips narrowband) in dark room at 22°C. Wild‐type leaves were treated similarly albeit without UV‐B radiation. One sample was frozen at the end of 6‐hr treatment. The second and third samples were transferred to the darkness and white light (100 μmol m^−2^ s^−1^), respectively, for 24 hr. Samples were snap‐frozen and stored at −80°C till DNA extraction and thymine dimer determination.

The UV‐B‐induced thymine dimers were quantified as described by Stapleton, Mori, and Walbot (1993). Six micrograms of genomic DNA isolated from UV‐B‐treated and control leaves was denatured, dot‐blotted onto Hybond N+ membrane (PerkinElmer life Sciences, Inc.), and baked at 80°C. The membrane was blocked, washed, and incubated with thymine dimer‐specific monoclonal antibodies (Sigma‐Aldrich, 1:2000 in TBS) for 3 hr at room temperature with gentle agitation. The dimers were visualized by incubating with alkaline phosphatase‐conjugated secondary antibody (Sigma, 1:3000) and detection by nitroblue tetrazolium and 5‐bromo‐4‐chloro‐3‐indolyl phosphate reaction. The bands were quantified by densitometry using ImageJ software. The thymine dimers were quantified using three independent biological replicates. However, in the figures, a single representative blot and the values derived from it using ImageJ software are shown.

### 4.7 Meiotic chromosome preparation

Floral buds at the appropriate stages of the meiosis were picked according to Brukhin et al. (2003). The buds were fixed and stored in Carnoy's solution [ethanol: chloroform: glacial acetic acid (6:3:1)] at 4°C or −20°C. Fixed inflorescences were rinsed with ethanol: glacial acetic acid (3:1) at defined intervals. Procedure for meiotic chromosome preparation was same as described by Ross et al. (1997). Chromosomes were counterstained with AT‐specific fluorescent dye, 4′‐6‐diamidino‐2‐phenylindole (DAPI, 1 μg/ml) (Sigma Laboratories). All images were captured using a 63× (1.4 N.A.) oil‐immersion objective on a Leica SP5 confocal microscope. Images were further processed with Adobe Photoshop CS6.

### 4.8 Mitotic chromosome preparation

Roots tips (1.5–2 cm) from 5‐day‐old seedlings were fixed in Carnoy's solution for 24 hr followed by washing with 70% (v/v) ethanol (15 min. 3×) and storage at 4°C. Procedure for mitotic chromosome preparation was same as described by Babich, Segall, and Fox (1997). Then, the tips were observed at 100× under Nikon compound microscope (Nikon Eclipse 80i) and photographed (Nikon NIS elements Basic version 3.1). Root tip nuclei were also assessed for nuclear size in somatic tissue by fixing in ethanol:acetic acid (3:1) fixative for at least 1 hr, cleared in 70% ethanol, and stained with 1 μg/ml 4′,6‐diamidino‐2‐phenylindole (DAPI) solution.

### 4.9 Immunofluorescence

For immunofluorescence, synaptonemal complexes were prepared as described by Ross et al. (1997) and Chelysheva et al. (2010); 60% (v/v) acetic acid treatment was performed at 45°C for 5 min with continuous stirring with a hook to remove maximum cytoplasm debris. Chromosomes were counterstained with DAPI and observed under the microscope to ensure proper slide preparation. The slides were microwaved in 10 mM citrate buffer pH 6 for 45 s at 850 W for proper fixing of the chromosomes to slides. Slides were first immersed in 0.1M phosphate‐buffered saline (PBST): pH 7.4 containing 0.01% (v/v) Triton X‐100 for 2 × 5 min. To block nonspecific antibody binding, 100 μl of blocking buffer [1% (w/v) BSA in PBS] was applied directly to the slides; 100 μl of primary CENPC (centromere protein C) antibody (1:100) in blocking buffer [PBS + 0.1% (v/v) Triton + 1% (w/v) BSA] was applied directly to the slides, covered with parafilm, and incubated overnight at 4°C in a moist chamber. The slides were washed (2 × 5 min) with PBST before adding 100 μl of the secondary antibody. Secondary antibody, goat anti‐rabbit 488 (Molecular Probes; diluted 1:500), was incubated for 90 min at room temperature. Finally, the slides were washed for 2 × 5 min and mounted in DAPI (10 μg/ml) in Vectashield antifade mounting medium. Slides were examined using a 63× (1.4 N.A.) oil‐immersion objective on a Leica SP5 confocal microscope. Images were further processed with Adobe Photoshop CS6.

### 4.10 Fusion primer and nested integrated PCR (FPNI‐PCR)

To locate the unknown sequences flanking the insertion sites in the transgenic lines, FPNI‐PCR described by Wang et al. (2011) with few modifications was used. The primers used for the FPNI‐PCR are listed in Table S5. The cycling conditions were followed according to Wang et al. (2011). All PCR products were electrophoresed on a 2.0% (w/v) agarose gel and FPNI‐PCR products showing sizes more than 500 bp were chosen for sequencing. The purified PCR product was sequenced using Sanger sequencing (Macrogen, South Korea). The sequences were aligned to the tomato genome sequence (Sol Genomics Network, https://solgenomics.net/) using BLAST program.

## AUTHORS CONTRIBUTIONS

SS, AKP, MR, YS, and RS conceived and designed the experiments. SS and AKP performed the experiments. SS, AKP, KS, MR, YS, and RS analyzed the data. SS, MR, YS, and RS wrote the manuscript.
