# Supplementary material for: MutS‐Homolog2 silencing generates tetraploid meiocytes in tomato (Solanum lycopersicum)
Source: Plant Direct. 2018 Jan 2;2(1):e00017. doi: 10.1002/pld3.17 (PMC6508528; doi:10.1002/pld3.17)
Supplement: Supplementary file 1 [file PLD3-2-e00017-s001.pdf]

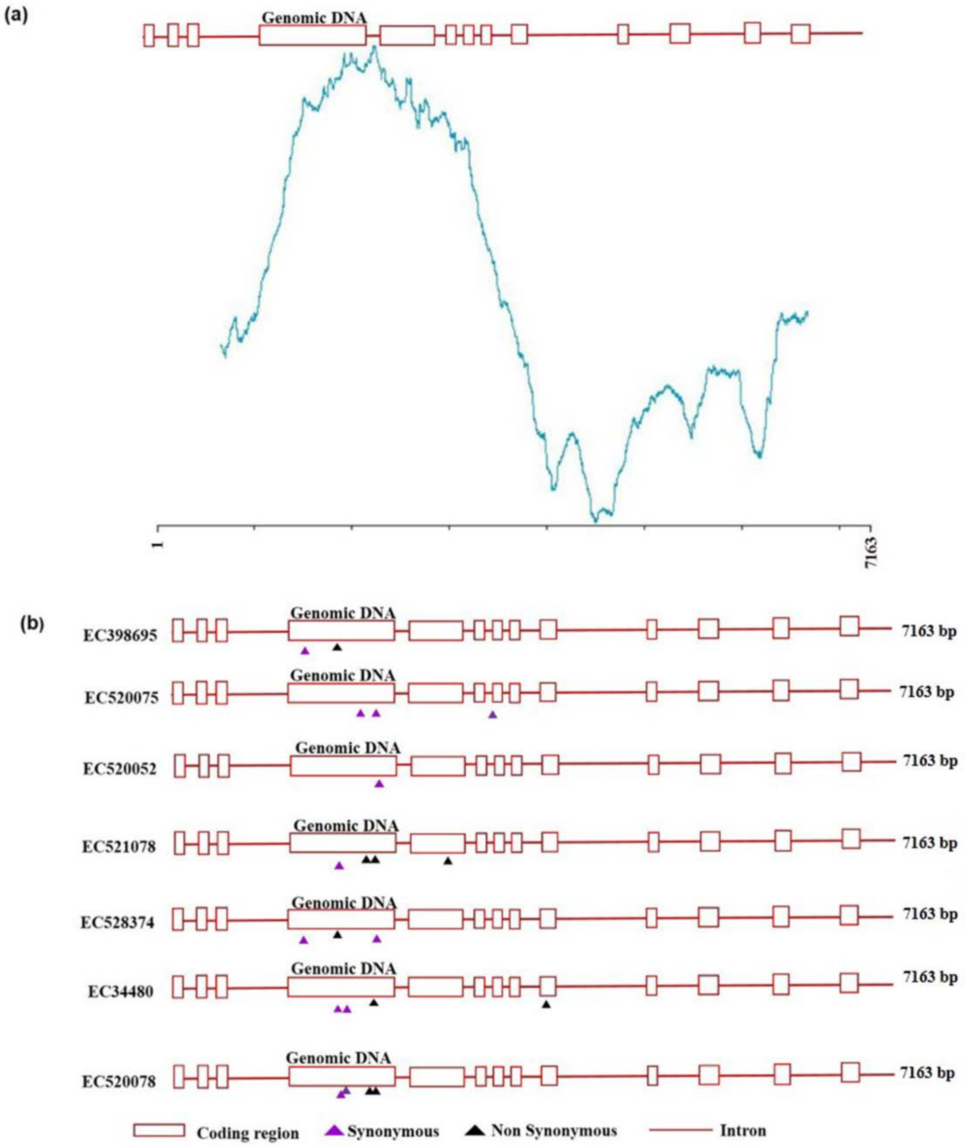


**Figure S1.** *In silico* prediction of most deleterious *MSH2* gene region by CODDLE **(a)** and distribution of SNPs detected in *MSH2* gene by EcoTILLING **(b). (a)** *In silico* prediction of most deleterious *MSH2* gene region by CODDLE. The tomato MSH2 gene consists of 7163 bp with 13 exons and 12 introns. The probability curve traced in blue represents the region of the gene where mutations would most likely deleteriously affect the function of encoded protein. Based on above prediction, the segment encompassing exons 3 to 9 was chosen for EcoTILLING. **(b)** Distribution of SNPs detected in *MSH2* gene by EcoTILLING. Red boxes denote exons interconnected with introns (solid red line). The black upright triangle indicates missense or nonsynonymous changes in the DNA sequence. The purple upright triangle indicates silent or synonymous changes. The numbers on the left and right of pictorial diagram respectively represent the accession number and total length of genomic DNA.

Figure S2. Multiple sequence alignment of mRNA sequences of tomato MSH family. Black box indicate the sequence of *MSH2* used for making *MSH2*-RNAi construct.

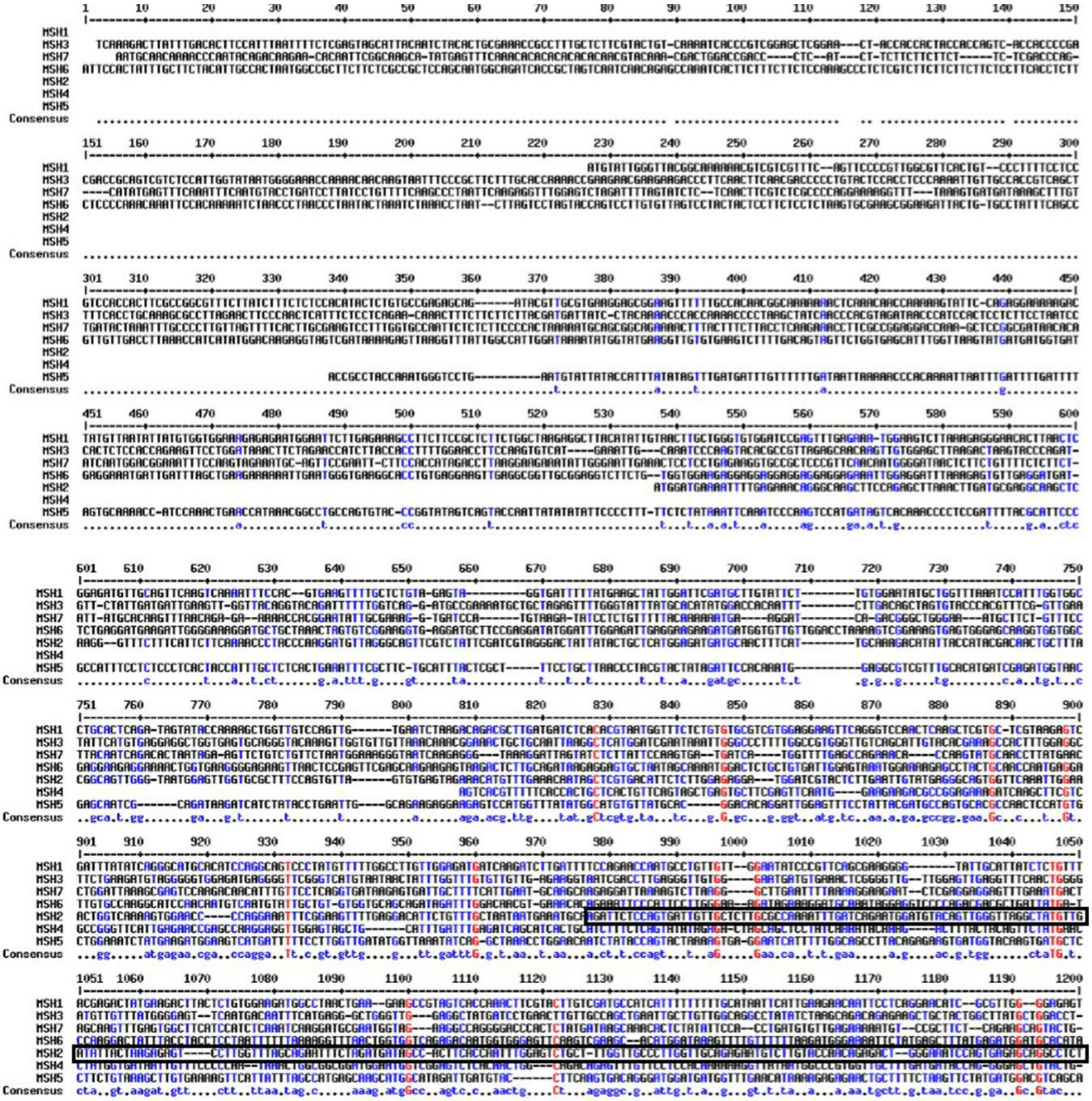

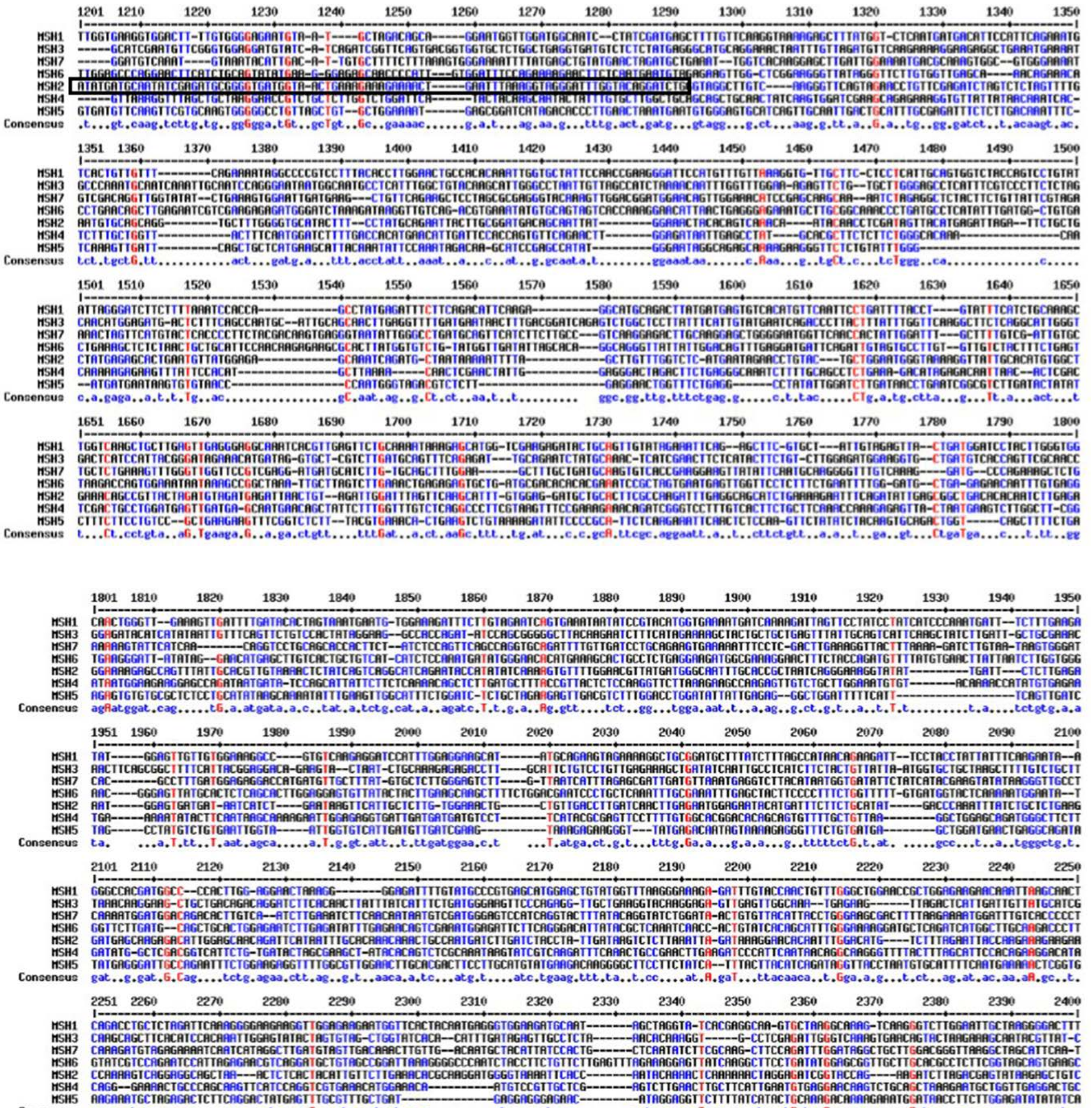

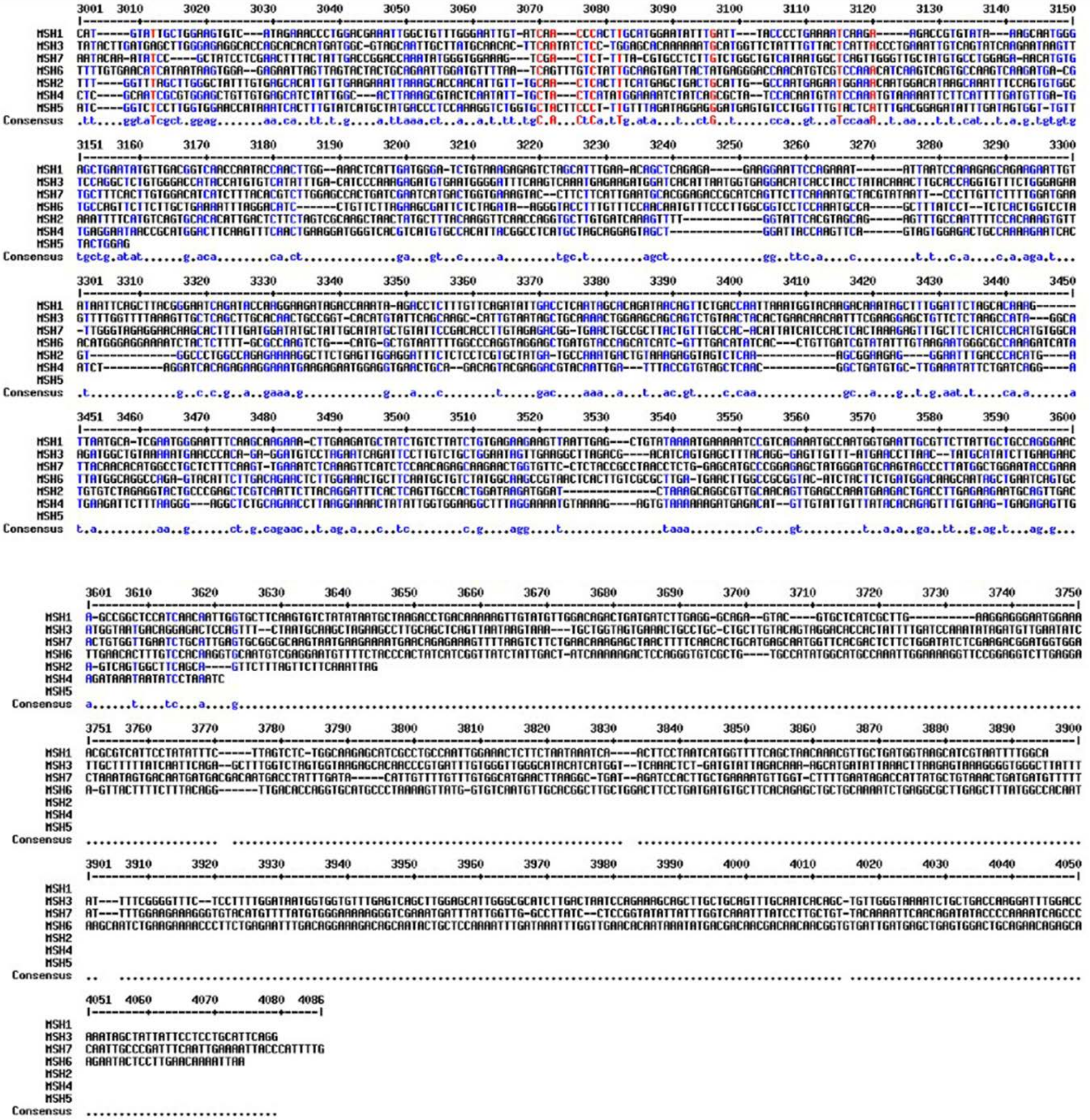
MSH1
MSH3
MSH7
MSH6
MSH2
MSH4
MSH5
Consensus

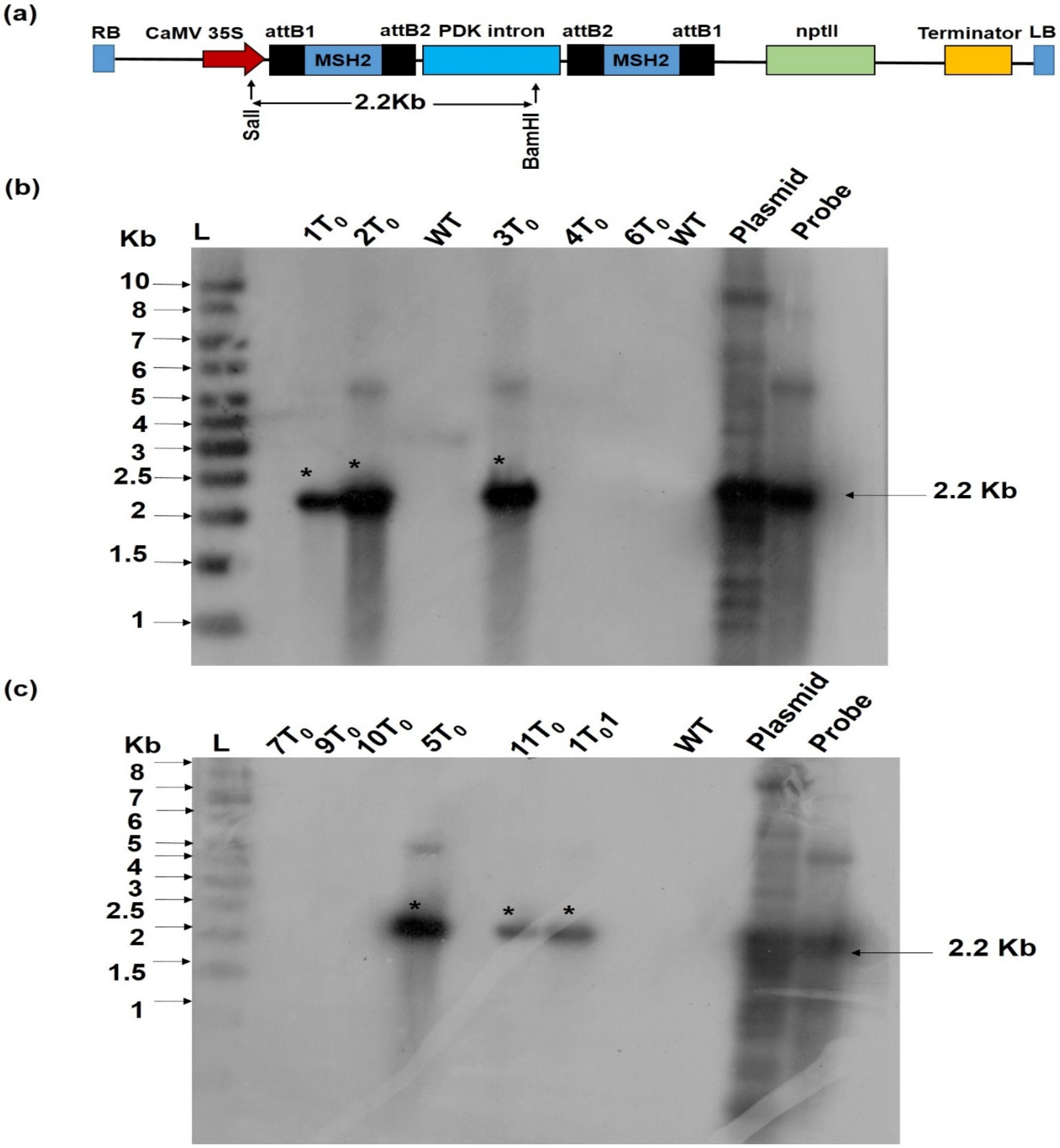


**Figure S3.** Generation of *MSH2*-RNAi transgenic tomato lines and Southern blot of $T_0$ transgenic lines. **(a)** Schematic representation of the construct used for *MSH2* silencing. The construct contains one spliceable intron with the targeted *MSH2* sequence forming a hairpin when the construct undergoes transformation. *AttB1* and *attB2* represent the two short stretches of sequences that participate in the recombination reaction of the Gateway system. T35S indicates 35S terminator. The restriction sites for BamHI and SalI are also indicated. **(b-c)** Southern blot of $T_0$ transgenic lines. Genomic DNA of $T_0$ plants was digested with BamHI and SalI to release the insert. The Southern blot was probed with radiolabelled *NPTII–NOS* probe of 2.2 Kb size. Numbers on the top of lanes $1T_0$ to $6T_0$ in panel (**b)** and lanes $7T_0$ to $1T_0$1 in panel **(c)** indicate the plant number of respective $T_0$ lines. **L-** 1 Kb DNA Ladder (Fermentas). **WT-** Wild type genomic DNA (negative control); **Plasmid-,** Plasmid DNA bearing *MSH2*-RNAi construct. **Probe-** Radiolabelled NPTII–NOS 2.2 Kb probe. Note: The *NPTII–NOS* probe was obtained by digesting *MSH2*-RNAi plasmid with BamHI and SalI. The asterisk (*) indicates the presence of *NPTII–NOS* sequence in the transgenic lines.

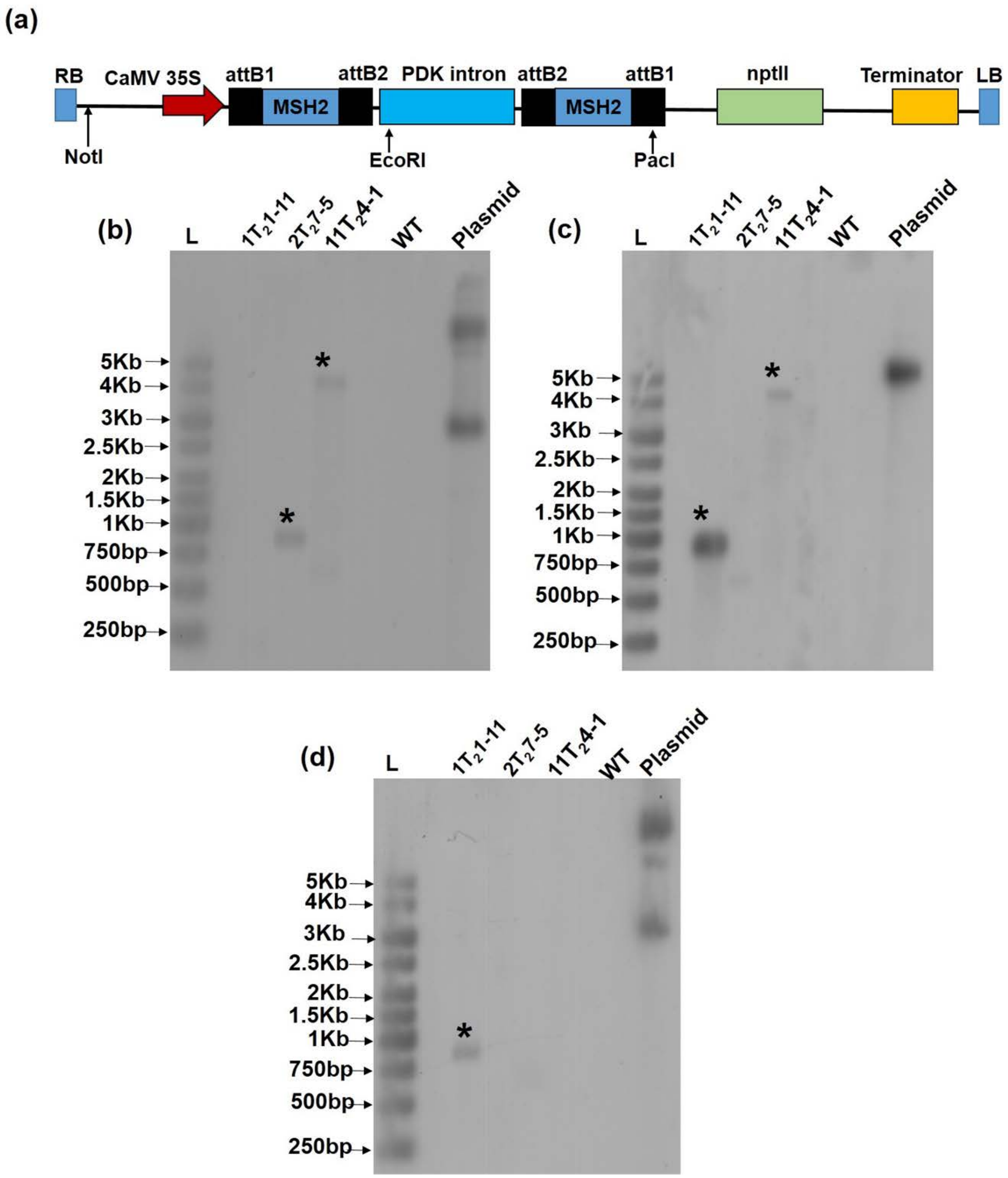


**Figure S4.** Southern blot of T$_2$ *MSH2*-RNAi lines 1T$_2$1-11, 2T$_2$7-5 and 11T$_2$4-1. **(a)** Schematic representation of the construct used for *MSH2* silencing. Black arrow represents the site of the restriction enzyme used for digesting Genomic DNA in Blot **(b), (c)** and **(d).** DNA was digested with EcoRI **(b),** with NotI **(c),** and PacI **(d). L**-1 Kb DNA Ladder (Fermentas). **WT-** Wild-type genomic DNA (negative control); **Plasmid-**, Plasmid DNA bearing *MSH2*-RNAi construct. The blots were probed with radiolabelled *NPTII* sequence. The asterisk (*) indicates the presence of *NPTII* sequence in the transgenic lines.

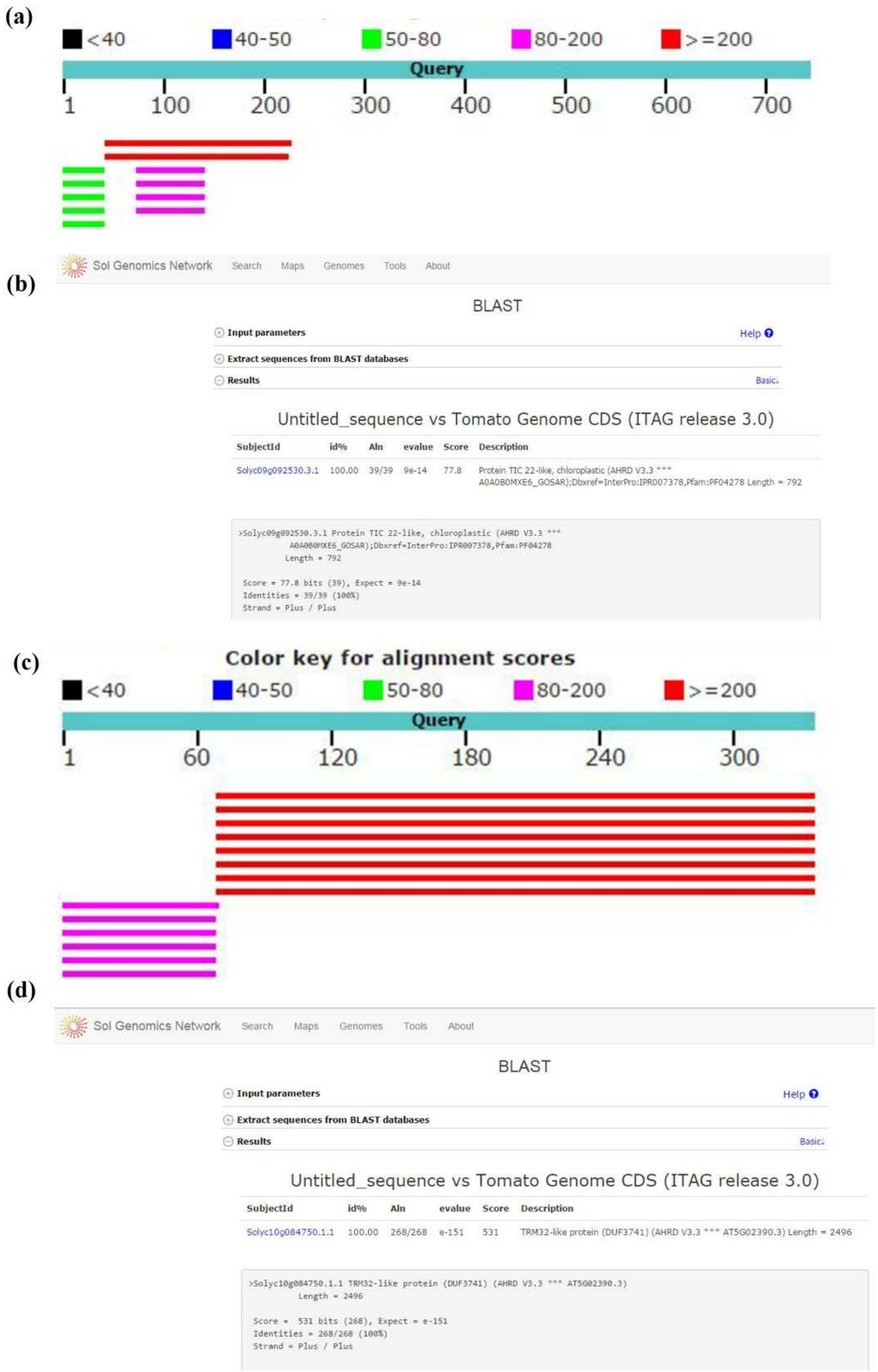


**Figure S5.** Sequence analysis of FPNI-PCR product of Line No. 1T$_2$1-11 and 2T$_2$5-5. The FPNI-PCR product from Line 1T$_2$1-11 showed 98% homology to tomato chromosome 9 in NCBI gene database **(a)** to *Tic22* gene of tomato in SGN database **(b).** The FPNI-PCR product from Line 2T$_2$ 5-5 showed 100% homology in chromosome 10 in NCBI database **(c)** and to *TRM32* gene of tomato in SGN database **(d).**

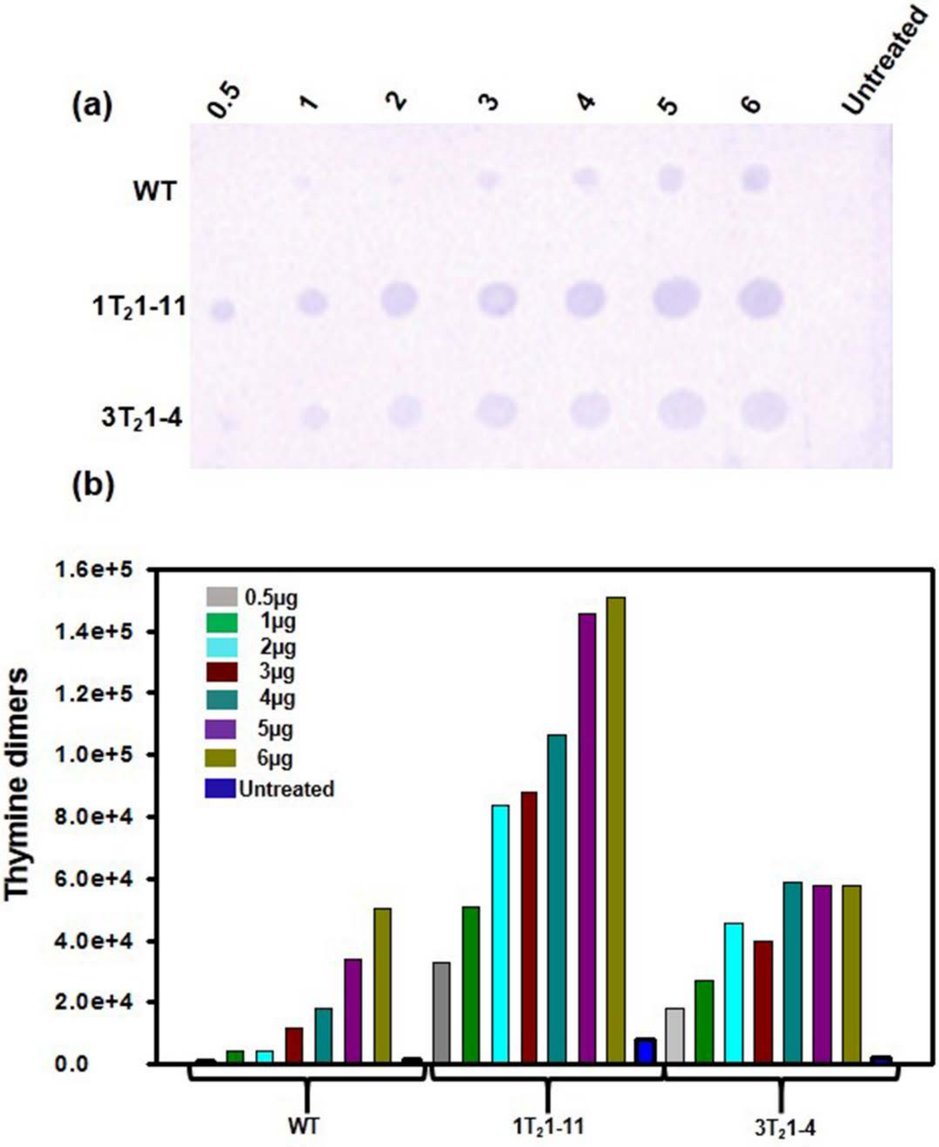


**Figure S6.** Quantification of thymine dimer levels in genomic DNA of WT and UV-B-treated *MSH2*-RNAi lines, $3T_21$-4 and $1T_21$-11. **(a)** Quantification of thymine dimer with different dilutions of genomic DNA. Numbers on top of the blot indicate the spotted amount of genomic DNA (µg) and on the left indicate plant number. **(b)** Quantification of thymine dimer formation in *MSH2*-RNAi lines in **(a)** by ImageJ analysis.

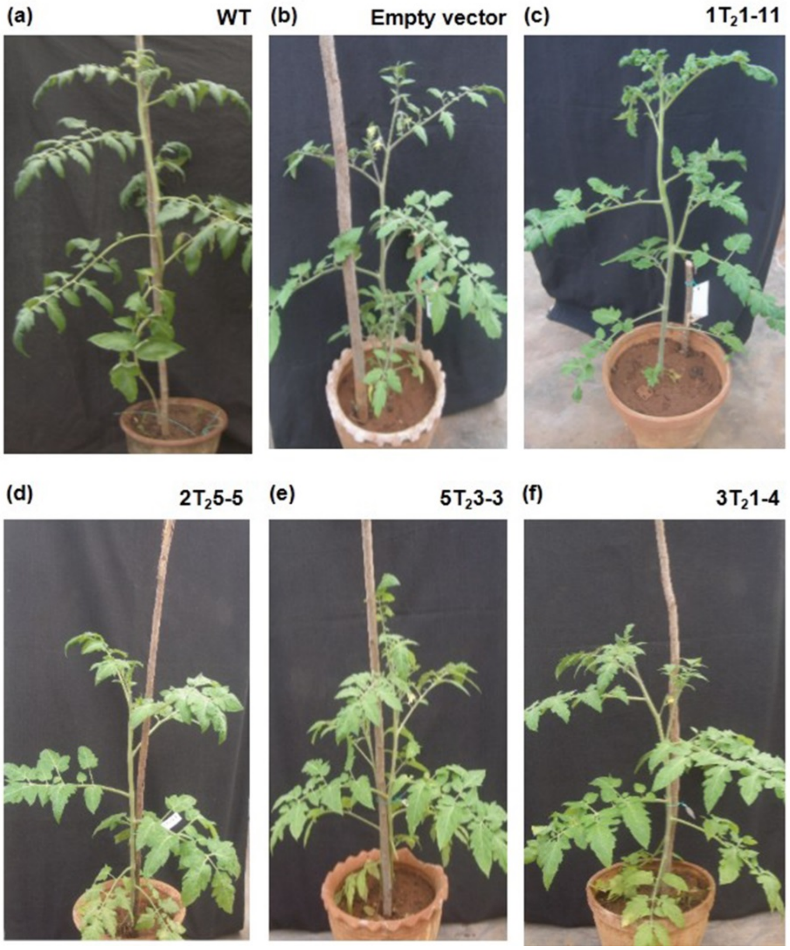


Figure S7- The vegetative morphology manifested by different *MSH2*-RNAi $T_2$ Plants.

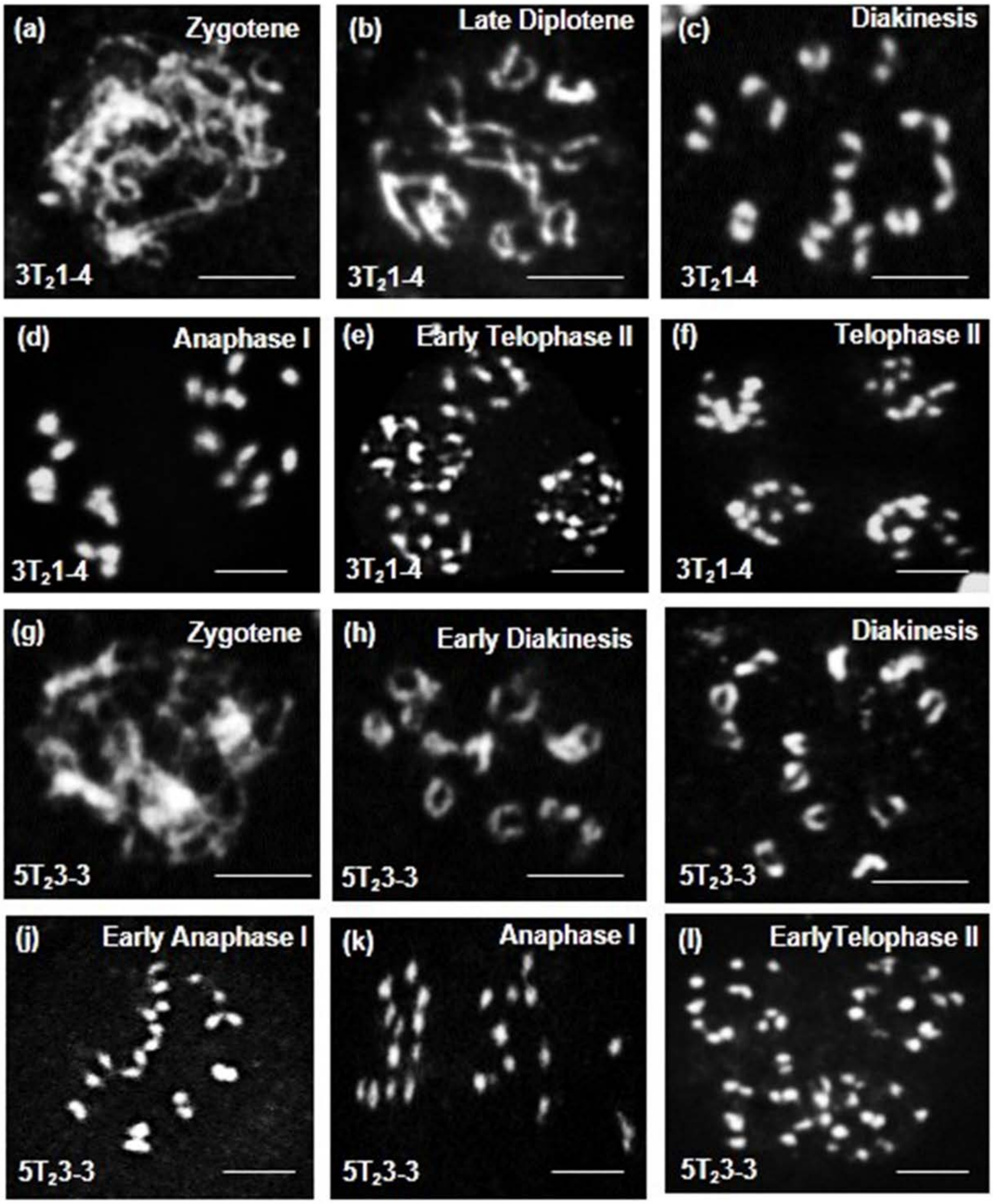


**Figure S8.** Male meiosis in *MSH2*-RNAi tomato lines. Representative meiotic stages of $3T_2$1-4 **(a–f)** and $5T_2$3-3 **(j-q)** lines from zygotene to telophase. The $3T_2$1-4 line with moderate *MSH2* silencing and $5T_2$3-3 line with no *MSH2* silencing displayed diploid meiotic chromosomes. Scale bar, 10 µm.

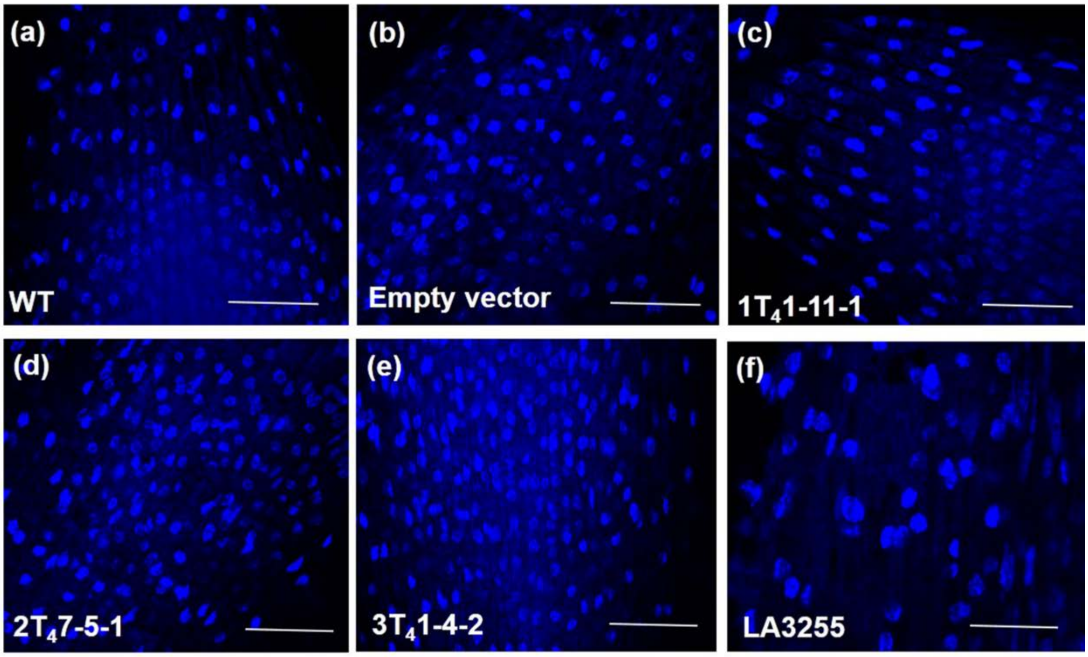


**Figure S9.** DAPI staining of root tip nuclei of WT and different *MSH2*-RNAi lines. **(a)** WT, **(b)** empty vector control, **(c-e)** progenies of $T_3$ *MSH2*-RNAi lines- $1T_21$-11-1 **(c),** $2T_27$-5-1 **(d),** $3T_2$ -4-2 **(e)** and LA3255 **(f).** Tetraploid tomato line LA3255 shows distinct enlarged tetraploid root tip nuclei in comparison to normal diploid nuclei in WT, control and *MSH2*-RNAi lines. Scale Bar, 50 µm.
