# Supplementary material for: MutS‐Homolog2 silencing generates tetraploid meiocytes in tomato (Solanum lycopersicum)
Source: Plant Direct. 2018 Jan 2;2(1):e00017. doi: 10.1002/pld3.17 (PMC6508528; doi:10.1002/pld3.17)
Supplement: Supplementary file 2 [file PLD3-2-e00017-s002.docx]

**Table S1**. Polymorphisms in 2.8 Kb region of *MSH2* gene in 54 tomato accessions analyzed by Aflitos et al. (2014). Out of 54 accessions SNPs were found only in ten accessions, indicating low frequency of polymorphism in this gene. (Aflitos, S et al. 2014 "Exploring genetic variation in the tomato (*Solanum section Lycopersicon*) clade by whole‐genome sequencing." *The Plant Journal* 80: 136-148).

| **Accession No.** | **No. of SNPs** | **Base Change** |
| --- | --- | --- |
| LA1421 | 3 | A42992380C |
|  |  | A42993608C |
|  |  | T 42993764A |
| Rote Beere | 4 | T42991683A |
|  |  | A 42991978G |
|  |  | A42992380C |
|  |  | A 42993608C |
| Jersey Devil | 1 | G42993054C |
| Gardeners Delight | 4 | T42991683A |
|  |  | A42991978G |
|  |  | A42992380C |
|  |  | A42993608C |
| LYC2962 | 3 | A42992380C |
|  |  | C42992796T |
|  |  | A42994119G |
| Katinga Cherry | 2 | T42991683A |
|  |  | A42993608C |

| **Accession No.** | **No. of SNPs** | **Base Change** |
| --- | --- | --- |
| *S. lycopersicum* Cultivar 1 | 8 | G42991669A |
|  |  | A42992380C |
|  |  | A42992595G |
|  |  | T42993167C |
|  |  | A42993608C |
|  |  | T42993764A |
|  |  | A42994119G |
|  |  | C42994316A |
| *S. lycopersicum* Cultivar 2 | 4 | T42991683A |
|  |  | A42991978G |
|  |  | A42992380C |
|  |  | A42993608C |
| *S. lycopersicum* Cultivar 3 | 8 | G42992331A |
|  |  | C42992309T |
|  |  | A42992380C |
|  |  | A42992595G |
|  |  | T42992928A |
|  |  | T42993167C |
|  |  | A 42994119G |
|  |  | C42994316A |
| *S. lycopersicum* Cultivar 4 | 1 | G 42992331A |

**Table S2**: The gene sequences flanking right border of T-DNA obtained using FPNI-PCR. Bold lowercase letters indicate bacterial right border T-DNA sequence and uppercase letters indicate flanking genome sequence from transgenic *MSH2*-RNAi plants.

| **Line No.** | **Right Border T-DNA sequence** | **FNPI-PCR sequence data** |
| --- | --- | --- |
| 1T_2_1-11 | RB | **gtttattagaataatcggatatttaaaagggcgtgaaaaggtaaatgacaactatacatctgattgatatttatttacagtcgaggagcttaattctaagaa**GCCAAAACAAGAGATATCGGCCTGTTTTCTTCAGGAAGGTAAGCTAATTGTCTTGAGTTAGTATCCACTATCCACTAGTGTGATACAATGCTATATCTCCCTTCTTGAAATTTCGTCAACTTCTGACTCGCACACCACGCGTGCCCTATAGTAGAAAGTCGTATTACAACTTTTGATTCGTACACCACGCGTGCCCTATAGTAAGTCGTTTCTCACCTCCTCCGACGTAATAAAGCAAGACTCAATCCCAACGACTATAAGAAGAAGCGAACTGAATTGTCCAGACATAACCTAGAAACAATTTTAGTTGAGAATGGAGCGCATTCACTACAGTCACGATCCAGCCCAGCAGAGAGAGTAGAGGCGGAGCCAGCATACGACATCACAATCAATTTAATTGTCTATGGACTCGGTTTGGACGGTTGTAAGAACTCGGAGGTGGAGGGGGGGAGGCTGACTTAGGACCTATTCACACTGAAAGAAACATTACTGTTTCTGTGGTCTTTATTATTCGTCTATCAGCTCTATAGTACTTGAGAGAAGGGATGACTGGACTGCCTCTGCTATCAGTTTTCGCGTAAGATTTACTTAGTATCGCTTCACCTGAGGTC GATCATGTGCCAACAGTCGAGGTCGTAAA |
| 2T_2_5-5 | RB | **gtttacccgccaatatatcctgtcaaacactgatagtttaaactgaaggcgggaaacgacaatctgatc**TCAGAAACACTCGATACAGCTGCTTCGACTGCATCTAGCAGCAACTTATATTCAGGGGCTACCAGGTCCAATGAGCAAAAGTCCCTGGATATTCCTTTAGGTTCAGAAAAGAAAACTCAACAAGATTCATGTTCAGACTCAAAGATTCTAGAAAATTCTCTTGATGTTAGCGAGAATTCCGATGACATTGGTGGCTTAAAGGCAGAGGAGAACTCTTTTCCAGTTGAATACAATATGGATGACAATTTATCTACTAACTCCACTTTGG |

**Table S3** List of genes and the primers used for screening for mutations and SNPs for the regions predicted by CODDLE based on genome sequence of Solanaceae Genome version 2.5. The *MSH2* gene information and location of the primers on genome sequence is also indicated.

| **Gene** | **Primer Sequence (5’-3’)** | | **Start Position** | **End Position** | **GC%** | **Tm** | **Amplicon size** |
| --- | --- | --- | --- | --- | --- | --- | --- |
| M13 | Fp | TGTAAAACGACGGCCAGT |  | | 50 | 53 |  |
|  | Rp | AGGAAACAGCTATGACCAT |  |  | 42 | 53 |  |
| *MSH2*  Solyc06g069230  (Set Ι) | Fp | TGTACCAATGTGCATTTTCTTCTT | 42994468 | 42994445 | 33 | 58 | 1243 bp |
|  | Rp | TAGCTAAGAAAAGAGGGGATTCAA | 42993248 | 42986063 | 38 | 60 |  |
| *MSH2*  Solyc06g069230  (Set ΙΙ) | Fp | CTGACACACAATCTTGAGAGGA | 42993225 | 42993378 | 45 | 60 | 914 bp |
|  | Rp | CAGTAGCACATCCAACTCAGAA | 42992510 | 42992489 | 45 | 60 |  |
| *MSH2*  Solyc06g069230  (Set ΙΙΙ) | Fp | CTTCTTTCCCAGCTCATTCATC | 42992585 | 42992563 | 45 | 60 | 972 bp |
|  | Rp | GGGGTGGACTTTTAAGACATCA | 42991635 | 42984452 | 45 | 60 |  |

**Table S4:** List of genes and the primers used for transcript analysis of MMR pathway.

| **Gene** | **Gene sequence id.** | **Primer Sequence (5ʹ->3ʹ)** | | **Start position** | **End position** | **GC%** | **Tm** | **Amplicon size** |
| --- | --- | --- | --- | --- | --- | --- | --- | --- |
| *MSH1* | [Solyc09g090870.2](http://solgenomics.net/feature/17939414/details)  (SGN) | Fp | GAGGAACTAAAGGGGAGATTTTGT | 70267311 | 70267288 | 42 | 62 | 136 bp |
|  |  | Rp | TGAATCTAGAGCAGGTCTGAGTTG | 70267199 | 70267175 | 46 | 63.6 |  |
| *MSH2* | Solyc06g069230 (SGN) | Fp | TTAGCTGGTTCACTTTCTGAGTTG | 42992524 | 42992501 | 42 | 62 | 102 bp |
|  |  | Rp | ATCTGGTGGACTGATATTTGGTCT | 42992446 | 42992425 | 42 | 62 |  |
| *MSH3* | XM_010319297  (NCBI) | Fp | TTCATAGAAAAGCTACTGCTGCTG | 1814 | 1837 | 42 | 62 | 100 bp |
|  |  | Rp | ACTTCTGTCCTCCGTAATGAAAAG | 1891 | 1914 | 42 | 62 |  |
| *MSH4* | XM_010326283  (NCBI) | Fp | AGAGATGAAGGAGACGGCTTTTAT | 1886 | 1909 | 42 | 62 | 139 bp |
|  |  | Rp | TAAGTGCCAATAGATGCTCACAAC | 2002 | 2025 | 42 | 62 |  |
| *MSH5* | XM_010329008  (NCBI) | Fp | GTAGACGTCTCTTGAGGAACTGGT | 1088 | 1111 | 50 | 65.2 | 119 bp |
|  |  | Rp | CACGTAAAGAGACCGAAACTTCTT | 1184 | 1207 | 42 | 62 |  |
| *MSH6* | [Solyc01g079520.2](http://solgenomics.net/feature/17683308/details)  (SGN) | Fp | AAGGTTTAACTGGTGGTCAGAGAC | 78582874 | 78582893 | 46 | 65.3 | 108 bp |
|  |  | Rp | GCATCCATCTCATAAAGCTCATAG | 78583173 | 78583196 | 42 | 62 |  |
| *MSH7* | XM_004242880  (NCBI) | Fp | GGATTTGCTTTTGTCGATTGTG | 1499 | 1520 | 41 | 58.4 | 116 bp |
|  |  | Rp | TATAACTTCCTTCGGTGACACTTG | 1592 | 1615 | 42 | 62 |  |

**Table S5:** List of genes and the primers used for FNPI-PCR. The common region is highlighted in bold.

| **Primer** | **Primer sequence (5ʹ-3ʹ)** | **Primer use** |
| --- | --- | --- |
| FP1 | **GTAATACGACTCACTATAGGGCACGCGTGGT** NTCGASTWTSGWGTT | 1st PCR primer |
| FP2 | **GTAATACGACTCACTATAGGGCACGCGTGGT** NGTCGASWGANAWGAA | 1st PCR primer |
| FP3 | **GTAATACGACTCACTATAGGGCACGCGTGGT** WGTGNAGWANCANAGA | 1st PCR primer |
| FP4 | **GTAATACGACTCACTATAGGGCACGCGTGGT** AGWGNAGWANCAWAGG | 1st PCR primer |
| FP5 | **GTAATACGACTCACTATAGGGCACGCGTGGT** NGTAWAASGTNTSCA A | 1st PCR primer |
| FP6 | **GTAATACGACTCACTATAGGGCACGCGTGGT** NGACGASWGANAWGAC | 1st PCR primer |
| FP7 | **GTAATACGACTCACTATAGGGCACGCGTGGT** NGACGASWGANAWGAA | 1st PCR primer |
| FP8 | **GTAATACGACTCACTATAGGGCACGCGTGGT** GTNCGASWCANAWGTT | 1st PCR primer |
| FP9 | **GTAATACGACTCACTATAGGGCACGCGTGGT** NCAGCTWSCTNTSCTT | 1st PCR primer |
| FSP1 | GTAATACGACTCACTATAGGGC | 2nd PCR primer |
| FSP2 | ACTATAGGGCACGCGTGGT | 3rd PCR primer |
| PRB1 | TTGACAGGATATATTGGCGGGT | 1st PCR primer |
| PRB2 | AAGGGCGTGAAAAGGTTTATC | 2nd PCR primer |
| PRB3 | CCATTTGTATGTGCATGCCAA | 3rd PCR primer |
